# Mechanistic role of GNE‐987 targeting BRD4‐HCP5 axis in pediatric T‐cell acute lymphoblastic leukemia

**DOI:** 10.1002/ccs3.70063

**Published:** 2026-02-14

**Authors:** Xu Sang, Mengying Jiang, Yanchun Guan, Xin Chen, Zhen Zhang, Yumeng Wu, Wansheng Peng

**Affiliations:** ^1^ Department of Pediatrics The First Affiliated Hospital of Bengbu Medical University Bengbu Anhui China

**Keywords:** Acute Lymphoblastic Leukemia, bromodomain‐containing protein 4, GNE‐987, HLA Complex P5, super‐enhancer, T‐cell acute lymphoblastic leukemia therapy

## Abstract

This study aims to explore the mechanism of action of the Bromodomain‐containing protein 4 (BRD4) inhibitor GNE‐987 in the treatment of pediatric T‐cell Acute Lymphoblastic Leukemia (T‐ALL), focusing on its effect in inhibiting T‐ALL cell proliferation by activating the HLA Complex P5 (HCP5) Super‐enhancer. Through bioinformatics approaches (including weighted gene co‐expression network analysis and least absolute shrinkage and selection operator regression analysis), key factor BRD4 was identified from the Gene Expression Omnibus database, along with its related regulatory genes and Super‐enhancer. In vitro experiments validated the regulatory effects of GNE‐987 on the expression of BRD4 and HCP5, and its impact on T‐ALL cell proliferation, colony formation, and apoptosis was assessed. Animal experiments further confirmed the efficacy of GNE‐987 in inhibiting T‐ALL progression by regulating HCP5. The results demonstrated that GNE‐987 significantly enhances the activity of the HCP5 Super‐enhancer and inhibits T‐ALL cell proliferation while promoting apoptosis by downregulating BRD4. This study suggests that BRD4 and HCP5 are potential therapeutic targets for T‐ALL, and GNE‐987 provides a novel therapeutic strategy by targeting this regulatory axis, laying the foundation for precision therapy in T‐ALL.

## Graphical abstract

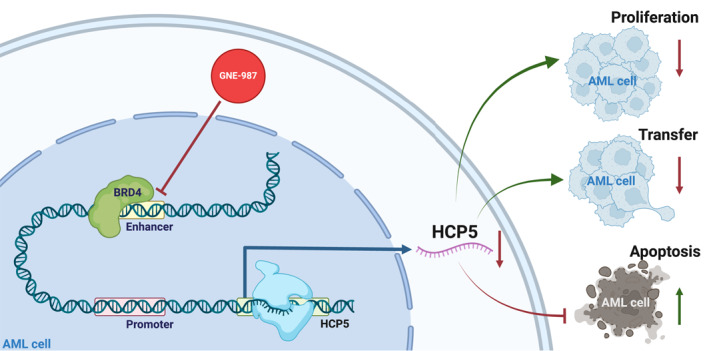

## 1 INTRODUCTION

T‐cell Acute Lymphoblastic Leukemia (T‐ALL) is a highly aggressive and fatal hematological malignancy that primarily affects children and adolescents.^1^ In T‐ALL, T lymphocytes undergo malignant transformation, leading to the abnormal proliferation of immature T lymphocytes in peripheral blood and bone marrow, accompanied by bone marrow suppression, severely impairing normal hematopoiesis. Patients typically present with severe anemia, thrombocytopenia, and recurrent infections. Furthermore, the high infiltration ability of T‐ALL can result in malignant cells invading multiple organs, such as the spleen, liver, and central nervous system, further complicating the disease and increasing the difficulty of treatment. Although modern chemotherapy has significantly improved the overall remission rate of T‐ALL, the relapse rate remains high, particularly in high‐risk pediatric patients, where drug resistance is becoming an increasingly prominent issue.^2^ The long‐term prognosis of T‐ALL remains poor, with survival rates for relapsed T‐ALL patients ranging from 20% to 40%. Additionally, existing treatment methods, such as hematopoietic stem cell transplantation and radiochemotherapy, are associated with significant toxic side effects, limiting their widespread clinical application.^3^ Therefore, identifying new molecular targets and developing more precise and less toxic treatment strategies have become priorities. Research on overcoming drug resistance in T‐ALL has also become a focal point in recent years.^4^, ^5^, ^6^

Among the numerous potential molecular targets, the epigenetic regulator Bromodomain‐containing protein 4 (BRD4), a member of the bromodomain and extra‐terminal domain (BET) family, has garnered widespread attention in recent years. BRD4, through binding to acetylated histones, participates in transcriptional regulation and chromatin remodeling, activating the expression of various cancer‐related genes and promoting the proliferation, survival, and migration of tumor cells.^7^, ^8^ In cancer research, BRD4 has been found to bind to super‐enhancer regions, enhancing the expression of oncogenes and thereby providing tumor cells with more robust proliferative and survival capabilities.^9^ BRD4 has been shown to play a tumor‐promoting role in various cancers, including breast cancer, prostate cancer, and medulloblastoma. In these cancers, BRD4 not only drives the overexpression of oncogenes through super‐enhancers but also promotes cancer cell survival and proliferation by inhibiting apoptotic pathways and enhancing cell cycle progression. However, despite extensive research on BRD4 in various solid tumors and hematologic malignancies, its role in T‐ALL still needs to be studied. Elucidating the specific regulatory pathways of BRD4 in T‐ALL will provide new insights into BRD4‐targeted therapies and may help address the current issue of drug resistance in T‐ALL treatment.^10^, ^11^

Super‐enhancers are specific genomic regions composed of densely clustered transcription factor binding sites that significantly enhance the expression of downstream target genes. Super‐enhancers are typically rich in acetylated histones, markedly boosting transcriptional activity, and are key regulatory elements that determine cell fate and maintain cellular characteristics. Increasingly, studies have shown that super‐enhancers play a particularly prominent role in cancer. Super‐enhancers are often associated with oncogenes driving cancer progression, and their hyperactivation can endow tumor cells with greater proliferative and survival abilities.^12^, ^13^ For example, the MYC gene expression driven by super‐enhancers is considered a critical oncogenic mechanism in various malignancies.^14^ Due to the high specificity and potent regulatory capacity, therapeutic strategies targeting super‐enhancers have garnered significant attention recently.^15^ The HLA Complex P5 (HCP5) super‐enhancer, a critical enhancer in several cancers, is closely associated with cancer development and progression.^16^ However, the function of the HCP5 super‐enhancer in T‐ALL remains unclear, and further research is needed to elucidate which essential genes and pathways it regulates in this context.

The development of BRD4 inhibitors has become a major focus in anti‐tumor drug research in recent years due to the critical role of BRD4 in cancer. GNE‐987 is a novel BRD4 inhibitor with promising anti‐tumor activity in multiple cancer models. By inhibiting the interaction between BRD4 and super‐enhancers, GNE‐987 effectively blocks the overexpression of oncogenes, suppresses tumor cell proliferation, and induces apoptosis.^17^ GNE‐987 demonstrates broad‐spectrum anticancer potential in solid and hematologic tumors, particularly showing strong inhibitory effects in tumors where BRD4 is highly active. Existing studies suggest that GNE‐987 exerts its effects by directly inhibiting BRD4 and suppressing the expression of related pathways and genes, significantly reducing tumor cell viability.^18^ However, despite its significant effects on solid tumors, the precise mechanism of GNE‐987 in T‐ALL remains unclear. Specifically, further research is needed to understand how GNE‐987 regulates the HCP5 super‐enhancer in T‐ALL, to clarify its potential therapeutic value.^10^, ^19^

Given the crucial role of BRD4 in epigenetic regulation and the importance of super‐enhancers in tumor regulation, the primary aim of this study is to explore the mechanism by which the BRD4 inhibitor GNE‐987 regulates BRD4 through targeting the HCP5 super‐enhancer in T‐ALL. We assess the inhibitory effects of GNE‐987 on T‐ALL cells through both in vitro and in vivo experiments, verifying its molecular mechanisms in suppressing T‐ALL cell proliferation and promoting apoptosis by modulating the HCP5 super‐enhancer. This study reveals the regulatory axis of BRD4 and the HCP5 super‐enhancer in T‐ALL and validates the efficacy and safety of GNE‐987 as a potential therapeutic agent. Through this research, we aim to provide new molecular targets and therapeutic strategies for the precision treatment of T‐ALL and facilitate the clinical translation of BRD4 inhibitors. Further studies will also provide theoretical foundations for addressing resistance in T‐ALL treatment, helping to improve long‐term survival rates in T‐ALL patients.

## 2 MATERIALS AND METHODS

### 2.1 Public data download and preprocessing

First, we accessed the public database Gene Expression Omnibus (GEO) (https://www.ncbi.nlm.nih.gov/geo/) and downloaded the T‐ALL‐related transcriptome dataset GSE48558. We randomly selected 12 samples, each from regular T and T‐ALL cells. Data download and initial processing were performed using *R* software (version 4.0.2) and packages GEOquery and ArrayExpress. The removeBatchEffect function from the limma package was used to eliminate batch effects to ensure consistency in subsequent analyses.

### 2.2 Weighted gene co‐expression network analysis (WGCNA)

We calculated each gene's Median Absolute Deviation (MAD) from the gene expression profiles and excluded the top 50% of genes with the smallest MAD. Then, we used the “goodSamplesGenes” method from the *R* package “WGCNA” to remove outlier genes and samples. Next, we used WGCNA to construct a scale‐free co‐expression network, setting the minimum gene dendrogram size to 50 and sensitivity to 11. Additionally, modules with a distance of less than 0.3 were merged, resulting in 3 co‐expression modules. Pearson correlation analysis (*p* < 0.05) was then used to assess the correlation between modules and groups. The gene module significantly associated with T‐ALL was selected as the disease‐related gene set for subsequent analyses.

### 2.3 Differential expression analysis

Differential expression analysis was performed using the DESeq2 package (version 1.28.1), with a significance threshold set at an adjusted *p*‐value less than 0.05. Differentially expressed genes were selected based on the |log_2_FoldChange| > 1 criterion, thereby identifying significantly upregulated or downregulated genes.

### 2.4 Least absolute shrinkage and selection operator (LASSO) regression algorithm

In our bioinformatics study, we employed the LASSO regression to identify key genes associated with the disease. First, random seeds were set to ensure experimental reproducibility, and the glmnet package was loaded to process the dataset with many variables. The glmnet function was used to perform LASSO regression on candidate disease‐related genes, modeling the data in a binary classification format, whereas the category was extracted as a response variable through regular expressions from the sample names. The model was evaluated by plotting the model object and using cv.glmnet for cross‐validation to determine the optimal lambda value. Finally, genes corresponding to nonzero coefficients extracted using the optimal lambda were considered key genes associated with disease status and were output. This method accurately identified vital genes and improved predictive accuracy by reducing model overfitting, effectively supporting biomarker discovery and disease mechanism research.

### 2.5 Venn diagram plotting, correlation analysis, and enhancer RNA (eRNA) acquisition

Venn diagrams were plotted using the online tool XianTao Academic, with eRNA data obtained from the literature. Correlation analysis was performed using chip data and analyzed through the SangerBox online platform.

### 2.6 Enrichment analysis

Differentially expressed genes were analyzed using GOKEGG enrichment analysis on the SangerBox platform, and a graphical visualization was generated.

### 2.7 Cell culture and grouping

The acute myeloid leukemia (AML) cell lines Tsuchiya Human Phagocyte‐1 (THP‐1) and Human Leukemia‐60 (HL‐60) (TIB‐202 and CCL‐240, ATCC), along with T‐ALL–specific cell lines Jurkat, CCRF‐CEM, MOLT‐4, and RPMI‐8402 (TIB‐152, CCL‐111, CRL‐1582, and CCL‐27, ATCC), were cultured in RPMI‐1640 medium (11875093, Thermo Fisher Scientific) supplemented with 10% fetal bovine serum (A5670701, Gibco) and maintained in an incubator at 37°C with 5% CO_2_. Cells were divided into control and experimental groups. The experimental group was treated with the BRD4 inhibitor GNE‐987 (HY‐129937A, MCE) at a final concentration of 20 nM, whereas the control group received an equal volume of DMSO (472301, Sigma‐Aldrich).^10^ The treatment duration was set to 24 h.

For the first part of the experiment, the cells were divided into the following two groups:

1. Ctrl group: treated with DMSO;
2. GNE‐987 group: treated with 20 nM GNE‐987 for 24 h.

For the second part of the experiment, the cells were divided into the following four groups:

1. Ctrl group: treated with DMSO;
2. GNE‐987 group: treated with 20 nM GNE‐987 for 24 h;
3. GNE‐987+sh‐NC group: cells stably transfected with sh‐NC lentivirus and treated with 20 nM GNE‐987 for 24 h;
4. GNE‐987+sh‐HCP5 group: cells stably transfected with sh‐HCP5 lentivirus and treated with 20 nM GNE‐987 for 24 h.

Before GNE‐987 treatment, cells were seeded in 6‐well plates (CLS3516, Corning) at a density of 1 × 10^6^ cells/mL. After 24 h, cell morphology was observed using an inverted fluorescence microscope (Axio Observer 7, ZEISS).^10^

### 2.8 Lentivirus construction

The lentiviral packaging service was provided by Sangon Biotech (Shanghai, China). The pHAGE‐puro series plasmids, along with the packaging plasmids pSPAX2 and pMD2.G, or the pSuper‐retro‐puro series plasmids with the helper plasmids gag/pol and VSVG, were co‐transfected into 293T cells (CRL‐3216, ATCC). After 48 h of cell culture, the supernatant was collected, filtered with a 0.45 μm filter, and centrifuged to collect the viral particles. The supernatant was collected again at 72 h, concentrated by centrifugation, and combined with the previous viral preparation. The viral titer was measured, and the overexpression efficiency was validated in the HL‐60 cell line (TIB‐71, ATCC, USA).

For lentivirus‐mediated cell transduction, 5 × 10^5^ cells were seeded in a 6‐well plate. When the confluency of the HL‐60 cells reached 70%–90%, an appropriate amount of lentivirus (MOI = 10, working titer approximately 5 × 10^6^ TU/mL) and 5 μg/mL polybrene (TR‐1003, Merck) were added to the medium for transduction. After 4 h of transduction, an equal amount of fresh medium was added to dilute the polybrene. The medium was replaced with fresh culture medium 24 h after transduction, and 48 h post‐transduction, the transduction efficiency was monitored using a luciferase reporter gene. Selection with 1 μg/mL puromycin (A1113803, Gibco) was applied to obtain stable cell lines. Once the cells no longer died in the puromycin‐containing medium, the cells were collected, and transduction efficiency was confirmed by reverse transcription‐quantitative polymerase chain reaction (RT‐qPCR).

### 2.9 RT‐qPCR

Total RNA was extracted from cells using the Trizol reagent kit (R0016, Beyotime). To synthesize cDNA, reverse transcription was performed using the reverse transcription kit (RR047A, Takara). The reaction system was prepared using the SYBR® Premix Ex Taq™ II kit (DRR081, Takara), and RT‐qPCR was performed on a real‐time quantitative PCR machine (ABI7500, Thermo Fisher). The PCR program was set as follows: initial denaturation at 95°C for 30 s, followed by 40 cycles of denaturation at 95°C for 5 s, and annealing at 60°C for 30 s. After the cycles, the program extended at 95°C for 15 s, 60°C for 60 s, and then 90°C for 15 s. The amplification curve was drawn. GAPDH was used as the internal reference, and all RT‐qPCR reactions were set up in triplicate wells, with the experiment repeated three times. The 2^−ΔΔCt^ method was used to calculate the relative expression levels of target genes in the experimental group compared to the control group. The formula is as follows: ΔΔCt = ΔCt (experimental group) ΔCt (control group), where ΔCt = Ct (target gene) − Ct (reference gene). Ct represents the cycle number at which the fluorescence intensity reaches the set threshold, indicating the exponential amplification phase. Primer designs are shown in Table 1.

**TABLE 1:** Primer sequences for reverse transcription‐quantitative polymerase chain reaction.

Gene name	Primer sequence
HCP5 (human)	Forward: 5′‐ GACTCTCCTACTGGTGCTTGGT ‐3′
Validated	Reverse: 5′‐CACTGCCTGGTGAGCCTGTT ‐3′
GAPDH (human)	Forward: 5′‐ TGCAACCGGGAAGGAAATGA ‐3′
Validated	Reverse: 5′‐ GCATCACCCGGAGGAGAAAT ‐3′

### 2.10 Western Blot

Total proteins were extracted from cells using RIPA lysis buffer (P0013B, Beyotime) supplemented with 1% PMSF (Phenylmethanesulfonyl fluoride), according to the manufacturer's instructions. The protein concentration of each sample was measured using a BCA kit (P0011, Beyotime) and adjusted to 1 μg/μL, with each sample having a total volume of 100 μL. The proteins were denatured by boiling at 100°C for 10 min, then stored at −80°C until further use. Depending on the size of the target protein band, an 8%–12% SDS‐PAGE gel was prepared. 50 μg of protein per sample was loaded into each lane for electrophoresis at a constant voltage (80 V initially, then 120 V) for 2 h. The proteins were transferred onto PVDF membranes (1620177, Bio‐Rad) using a constant current of 250 mA for 90 min.

The membrane was blocked at room temperature for 1 h using 5% nonfat milk in 1 × TBST, followed by washing with 1 × TBST for 10 min. The membrane was incubated overnight at 4°C with the primary antibody (Table 2 for antibody details) and washed three times with 1 × TBST, 10 min each time. Then, the membrane was incubated with HRP‐conjugated goat anti‐rabbit IgG (ab6721, Abcam, diluted 1:5000) or goat anti‐mouse IgG (ab205719, Abcam, diluted 1:5000) secondary antibodies at room temperature for 1 h, followed by washing three times with 1 × TBST for 5 min each. The membrane was incubated with ECL substrate (1705062, Bio‐Rad) for 1 min at room temperature and then exposed using the ImageQuant LAS 4000C imaging system (GE). GAPDH was used as the internal reference for total protein, and the relative expression level of the target protein was calculated as the ratio of the grayscale value of the target band to that of the reference band. Each experiment was repeated three times to determine protein expression levels.

**TABLE 2:** Antibody information for western blot.

Target name	Manufacturer	Catalog number	Dilution ratio
BRD4	Abcam	ab128874	1:1000
β‐actin (human)	Abcam	ab7817	1:1000

### 2.11 Chromatin immunoprecipitation quantitative polymerase chain reaction (ChIP‐qPCR)

The ChIP experiment used the SimpleChIP Enzymatic Chromatin IP Kit (#56383, Cell Signaling Technology, USA). DNA‐protein complexes in the fixed cells were immunoprecipitated using BRD4 antibody (ab128874, Abcam) and H3K27ac antibody (ab4729, Abcam). The extracted DNA was analyzed by qPCR using SYBR Green Master Mix (A57155, Thermofisher) on the StepOnePlus Real‐Time PCR System to assess the binding of BRD4 to the HCP5 super‐enhancer region and the modification levels of H3K27ac.

### 2.12 Cell counting kit‐8 (CCK‐8) assay

Cells were incubated in a 96‐well culture plate for 0, 12, 24, 36, and 48 h, with a cell density of 4 × 10^3^ cells/well. Before measurement, the cells in each group were washed with PBS and incubated with 100 μL of CCK‐8 solution (C0037, Beyotime) at 37°C for 1 h. Absorbance at 450 nm was measured using a microplate reader (Thermo Fisher Scientific, Inc.). Each independent experiment was performed in triplicate. Data are expressed as mean ± standard deviation.

### 2.13 Cell viability/death detection

To evaluate cell survival, we used the Live and Dead Assay Kit (L7012, Invitrogen). This assay utilizes calcein‐AM, which is retained in live cells and emits green fluorescence, whereas ethidium homodimer binds to nucleic acids in damaged cells and emits red fluorescence. Briefly, 1 × 10^6^ cells were treated according to the groups above, then stained using the Live and Dead Assay Kit (5 μM ethidium homodimer and 5 μM calcein‐AM) and incubated at 37°C for 30 min. Cells were analyzed using the Axio Vision 4.0 fluorescence microscope (Carl Zeiss Inc.).

### 2.14 Soft agar colony formation assay

A 1.2% agarose solution was autoclaved at 55°C. A double concentration of RPMI1640 medium was prepared, containing 20% FBS and a double concentration of penicillin and streptomycin. The medium was then filtered through a 0.2 μm membrane filter. To prepare the bottom layer gel, 0.75 mL of 1.2% agarose was mixed with 0.75 mL of double‐concentration medium and poured into each well, followed by overnight cooling. Subsequently, T‐ALL cells treated with various concentrations of GNE‐987 were washed with PBS and resuspended in a fresh medium at a concentration of 5 × 10^3^ cells/mL. For the top gel layer, 0.7% agarose gel was mixed with a double‐concentration medium at a 1:1 ratio, incubated, and the colony formation rate was calculated.

### 2.15 Flow cytometry analysis

The Annexin V‐FITC/PI kit (C1062L, Beyotime) was used to detect apoptosis in HL‐60 and THP‐1 cells treated according to the aforementioned groups. Cells were seeded in 6‐well plates, with 1 × 10^6^ cells per well. After collecting the cells, 195 μL of Annexin V‐FITC binding buffer was added to resuspend the cells, followed by 5 μL of Annexin V‐FITC solution and 10 μL of PI solution. The cells were incubated in the dark at room temperature for 15 min, and flow cytometry analysis was performed within 20 min using a BD FACS Calibur flow cytometer to assess apoptosis. The apoptosis rate was calculated as the sum of apoptotic cells in the Q1‐UR (upper right) and Q1‐LR (lower right) quadrants.

### 2.16 Construction of AML mouse model

Female BALB/c nude mice (purchased from the Animal Center of Nanjing Medical University) were housed under Specific Pathogen‐Free conditions and randomly assigned to the Ctrl group, GNE‐987 group, GNE‐987+sh‐NC group, and GNE‐987+sh‐HCP5 group. Each group of mice was injected with 200 μL of 2 × 10^6^ HL‐60 cells via the tail vein. 2 × 10^6^ sh‐NC or sh‐HCP5‐transduced HL‐60 cells were injected for the lentivirus transduction groups. Mice that required GNE‐987 treatment were injected with 100 μL of 0.2 mg/kg GNE‐987 ^20^ every two days. After 3 weeks of vaccination, three mice from each group were randomly sacrificed for sampling and experimental analysis. Human leukemia cells (hCD3+ cells) in bone marrow and peripheral blood were detected by flow cytometry. Hematoxylin and eosin (H&E) staining and immunofluorescence staining were performed on paraffin‐embedded spleen sections. The liver was collected post‐mortem and stored at −80°C for further analysis. All animal experiments were approved by the Experimental Animal Welfare Ethics Committee of The First Affiliated Hospital of Bengbu Medical University (approval number: 2024 No. 302).

### 2.17 Flow cytometry analysis of hCD3+ cells in peripheral blood and bone marrow

The mice were sacrificed 3 weeks post‐inoculation, and peripheral blood and bone marrow samples were collected. Bone marrow was extracted by flushing cold PBS through the femur and tibia, whereas peripheral blood was collected via the retro‐orbital plexus. Red blood cells were lysed using RBC lysis buffer (00‐4333‐57, Thermo Fisher Scientific), followed by washing twice with PBS containing 2% fetal bovine serum (26140‐079, Gibco, Thermo Fisher Scientific). After centrifugation (400 g, 5 min, 4°C), the cells were resuspended in PBS and stained with an anti‐human CD3‐APC antibody (OKT3 clone, 317312, BioLegend) at a dilution of 1:200. A total of 1 × 10^6^ cells were incubated with the antibody for 30 min at 4°C in the dark. After staining, the cells were washed with PBS and fixed with 2% paraformaldehyde (P6148, Sigma‐Aldrich) at room temperature for 10 min. Flow cytometric analysis was performed using a BD LSRFortessa™ flow cytometer (BD Biosciences). Data acquisition and analysis were performed using FlowJo software (v10.7.1, BD Biosciences, official website). The percentage of hCD3+ cells in peripheral blood and bone marrow was calculated by gating on single‐cell populations, excluding debris and doublets. At least 10,000 events were recorded for each sample. Each experiment was repeated three times.

### 2.18 H&E staining

Mice were anesthetized by intraperitoneal injection of Zoletil 50 (30 mg/kg) and sacrificed by cervical dislocation. Tissues were fixed with 4% paraformaldehyde for one day before performing H&E staining. The sections were first stained in hematoxylin solution (H3136, Sigma‐Aldrich) for 5 min to stain nuclei, followed by rapid differentiation in 1% acid alcohol. Next, sections were stained in eosin solution (230251, Sigma‐Aldrich) for 3 min to stain the cytoplasm and intercellular matrix. The sections were dehydrated twice in 95% ethanol, cleared, and mounted with neutral resin. Morphological images were observed and captured using a light microscope (Leica Microsystems, Germany). Image analysis was performed using ImageJ software (NIH, USA, version 1.52a) to quantify nuclei and cytoplasm and evaluate morphological characteristics. All steps were performed at room temperature, removing excess staining by washing the sections with distilled water after each step. At least three sections were prepared under each experimental condition, and each experiment was repeated three times to ensure reproducibility.

### 2.19 Immunofluorescence staining

After spleen excision, the tissues were fixed in 4% paraformaldehyde overnight, followed by dehydration in a graded series of alcohol, clearing, and paraffin embedding. Four‐micron‐thick sections were prepared and baked in an oven at 60°C. The tissue sections were then deparaffinized and rehydrated for antigen retrieval, followed by blocking with 3% BSA for 30 min. The sections were incubated overnight at 4°C with anti‐GRP78 antibody (ab21685, Abcam, 1:200) and anti‐Ki67 antibody (ab279653, Abcam, 1:200). Secondary antibodies, Alexa Fluor 647‐conjugated goat anti‐rabbit IgG (A0468, Beyotime) and FITC‐conjugated goat anti‐mouse IgG (A0568, Beyotime), were incubated for 60 min at 37°C in the dark. PBS washes (3 times, 3 min each) were followed by mounting with an antifade mounting medium containing DAPI (P0131, Beyotime). Images were captured using a laser confocal microscope (Zeiss LSM710).

### 2.20 Statistical analysis

Data were obtained from at least three independent experiments and are presented as mean ± standard deviation (Mean ± SD). For comparisons between the two groups, an independent sample *t*‐test was used. For comparisons among three or more groups, a one‐way analysis of variance (ANOVA) was applied. If ANOVA indicated significant differences, Tukey's HSD post‐hoc test was used to compare inter‐group differences. The Mann–Whitney *U* test or Kruskal–Wallis *H* test was employed for data with nonnormal distribution or unequal variance. All statistical analyses were performed using GraphPad Prism 9 (GraphPad Software, Inc.) and the R programming language. A *p*‐value <0.05 was statistically significant for all tests, with two‐tailed *p*‐values applied.

## 3 RESULTS

### 3.1 Bioinformatics analysis indicates BRD4 may be a key factor involved in T‐ALL

T‐ALL is an aggressive blood cancer that targets T lymphocytes in the body.^21^ It primarily affects children and adolescents and is less common in adults. Its treatment typically involves chemotherapy, sometimes combined with radiotherapy and targeted therapies, though these approaches are often of limited efficacy.^22^

**FIGURE 1:**
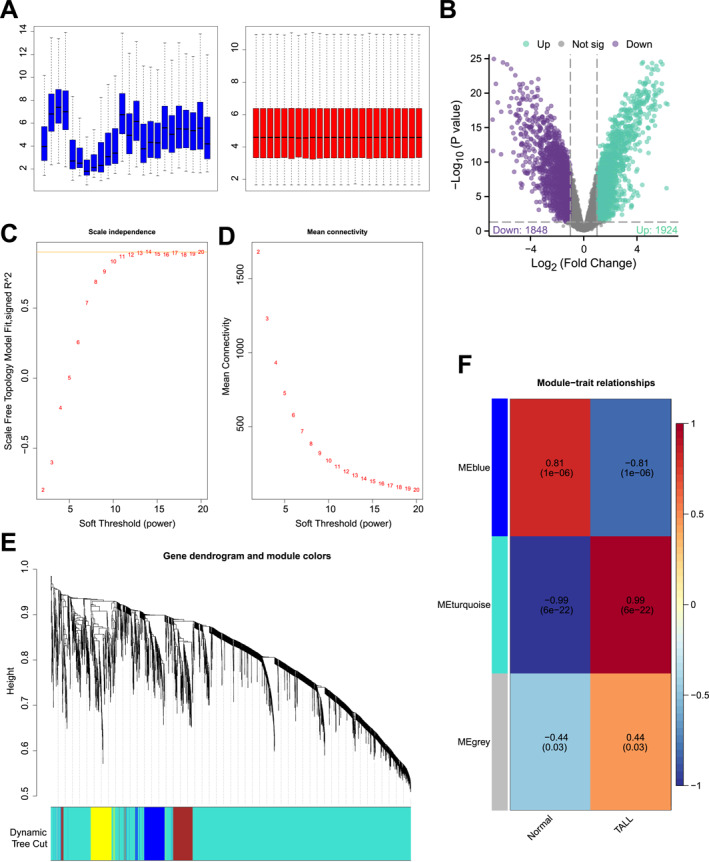
Bioinformatic Screening of Key Pathogenic Factors in T‐ALL. (A) Correction of batch effects in the sample dataset; (B) volcano plot of differentially expressed genes between normal T cells and T‐ALL cell lines, with downregulated genes shown in purple, upregulated genes shown in green, and genes without significant differential expression shown in gray; (C, D) scale independence, mean connectivity, and scale‐free topology plot, with the weighted value *β* = 11 selected to meet the scale‐free network criterion; (E) dendrogram of co‐expression network modules; (F) correlation between gene modules obtained through clustering and normal T cells versus T‐ALL cell lines. The number of samples in each group, *n* = 12. T‐ALL, T‐cell Acute Lymphoblastic Leukemia.

To explore potential therapeutic targets for T‐ALL and develop new treatments, we searched for T‐ALL‐related transcriptome datasets in the GEO database and selected the GSE48558 dataset to investigate the molecular mechanisms of the disease. After correcting for batch effects (Figure 1A), we conducted differential expression analysis, identifying 3772 genes that were significantly differentially expressed between normal T cells and T‐ALL cells. Among these, 1848 genes were significantly downregulated, whereas 1924 genes were significantly upregulated (Figure 1B).

To further identify disease‐related genes, we performed WGCNA. WGCNA is a widely used and powerful method in bioinformatics for identifying disease‐causing genes by constructing co‐expression networks from gene expression data. This method identifies key gene modules related to the disease, providing insights into the molecular mechanisms of the disease and aiding in the screening of critical pathogenic genes.^23^ Using *R* software, we calculated the network's soft threshold power (*β*) and determined that the optimal *β* value for satisfying the scale‐free network criterion was 11 (Figure 1C–D). Based on this soft threshold, we set the minimum module size to 50 and the module merging threshold to 0.3, followed by dynamic hybrid cutting (Figure 1E). The results identified three gene modules: MEturquoise, MEblue, and MEgrey. Using these three modules, we classified the normal and T‐ALL samples according to their clinical traits and further analyzed the correlation between gene modules and clinical traits. The results showed that the MEblue module was most negatively correlated with T‐ALL, with a correlation coefficient of −0.81 and a *p*‐value of less than 0.001. The MEturquoise module was most positively correlated with T‐ALL, with a correlation coefficient of 0.99 and a *p*‐value of less than 0.001 (Figure 1F).

To further screen for critical factors, we selected the genes from the MEblue module, which had the strongest negative correlation with T‐ALL. A regression analysis model was applied to the module genes for fitting analysis to remove genes with similar features. We used the glmnet function to perform LASSO regression on the module genes. Using a regular expression, we modeled the data as a binary classification problem and extracted the category from the sample names as the response variable. The model was evaluated by plotting the model object and using cv.glmnet for cross‐validation to determine the optimal lambda value (Figure S1A). Ultimately, the genes corresponding to nonzero coefficients extracted with the optimal lambda value were considered key genes associated with disease status. The final result identified three feature factors: TXNRD1, BRD4, and MT‐TS1 (Figure S1B). According to the literature, BRD4 is a critical transcriptional regulatory protein influencing cell proliferation and survival by recognizing acetylated histones and promoting gene expression. In malignancies such as leukemia, the overexpression and dysfunction of BRD4 are closely related to the formation of pathological conditions,^24^ consistent with our findings (Figure S1C).

These results suggest that BRD4 may be a critical factor in T‐ALL progression.

### 3.2 STK3 is a key BRD4‐regulated eRNA in T‐ALL

**FIGURE 2:**
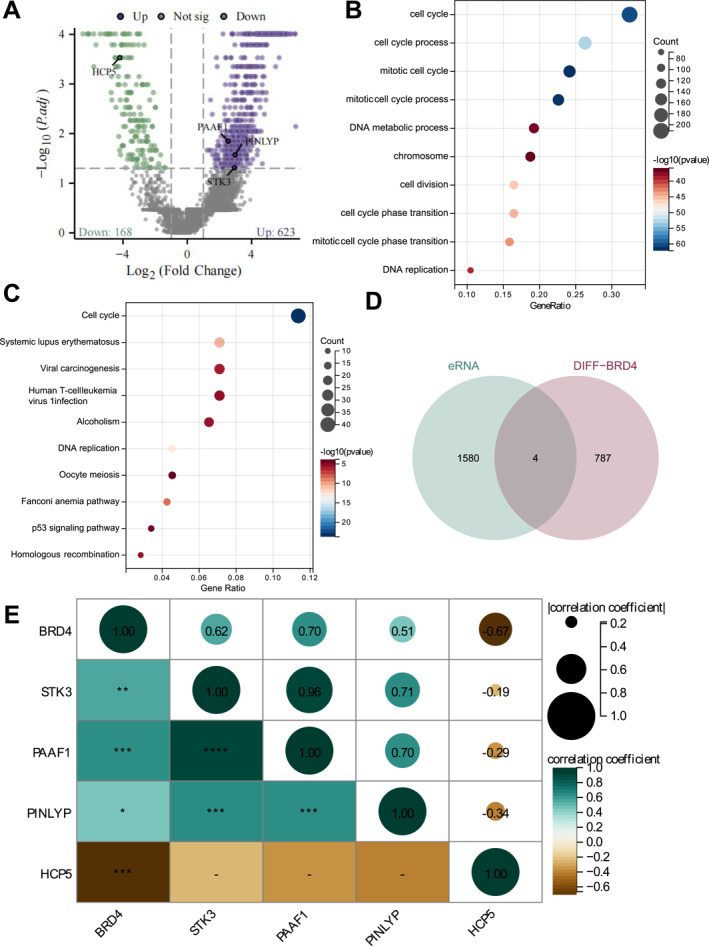
Identification of Key eRNA regulated by BRD4. (A) Volcano plot of differential expression between high and low BRD4 expression groups. (B) GO enrichment analysis of differentially expressed genes. (C) KEGG enrichment analysis of differentially expressed genes. (D) Venn diagram showing the intersection of differentially expressed genes and eRNA. (E) Correlation analysis between BRD4 and HLA Complex P5, STK3, PAAF1, and PINLYP expression. BRD4, Bromodomain‐containing protein 4.

BRD4 is a critical protein involved in regulating gene transcription. It recognizes acetylated histones via its bromodomain, recruiting transcriptional machinery to chromatin, thereby modulating gene expression and the cell cycle.^24^ To investigate the specific regulatory mechanism of BRD4 in T‐ALL, we categorized samples into high and low‐expression groups based on the median expression value of BRD4 in transcriptomic data. Differential expression analysis was then performed to identify BRD4‐related genes, yielding 791 genes closely associated with BRD4 (Figure 2A, Table S1). Further enrichment analysis revealed the main pathways regulated by BRD4 in T‐ALL. GO enrichment analysis showed that differentially expressed genes between high and low BRD4 expression groups were significantly enriched in biological processes such as “Cell Cycle,” “Mitotic Cell Cycle,” and “DNA Metabolic Process” (Figure 2B), suggesting that BRD4 plays a pivotal role in regulating the proliferation and cell cycle progression of T‐ALL cells. KEGG enrichment results indicated that these genes were enriched in pathways such as “Cell Cycle,” “Systemic Lupus Erythematosus,” “Viral Carcinogenesis,” and notably, “Human T‐cell Leukemia Virus Type 1 Infection” (HTLV‐1), which is directly related to T‐ALL (Figure 2C). These results suggest that BRD4's differential expression may influence T‐ALL's pathogenesis and progression through the cell cycle regulation and multiple immune escape‐related signaling pathways.

Recent studies have shown that BRD4's regulatory functions are not limited to direct action on gene promoter regions. It is also enriched at enhancers, particularly Super‐enhancers, influencing gene expression by regulating these regions' activity.^24^ eRNA are noncoding RNA molecules transcribed from active enhancers, and they have been demonstrated to play important roles in gene expression regulation, chromatin conformation, and the three‐dimensional structure of the genome.^25^ Dynamic gene expression regulation is crucial to leukemia cell growth and survival during T‐ALL progression.^26^ To further understand how BRD4 regulates distant gene expression through enhancers and to explore potential therapeutic targets, we screened for eRNA regulated by BRD4. By intersecting the 791 BRD4‐related genes with the obtained eRNA data, we identified four overlapping factors: HCP5, STK3, PAAF1, and PINLYP (Figure 2D). Correlation analysis was conducted to explore their relationship with BRD4, showing that STK3, PAAF1, and PINLYP were positively correlated with BRD4 expression, whereas HCP5 was negatively correlated (Figure 2E). Moreover, based on the obtained eRNA data, HCP5, STK3, and PINLYP all exhibited multiple enhancer sites, displaying Super‐enhancer characteristics, suggesting that they may play critical roles in T‐ALL through Super‐enhancer mechanisms.^27^ Literature reports indicate that HCP5 is an endogenous retrovirus‐associated gene that may regulate noncoding RNA or be involved in immune escape mechanisms in certain cancers.^28^ STK3 is a gene closely associated with apoptosis and cell proliferation. It can suppress tumor growth by inducing apoptosis, regulating the PI3K/AKT/mTOR pathway, and affecting cell cycle progression and motility.^29^ PAAF1 acts as a negative regulator of proteasome activity through interaction with proteasomal ATPases.^30^ PINLYP, containing an LY6/PLAUR domain, has phospholipase A2 inhibitory activity. Studies have shown that PINLYP can modulate type I interferon responses, thereby playing an important role in antiviral immunity and immune homeostasis.^31^ Given the close association between T‐ALL and the immune system, we identified HCP5 as the key BRD4‐regulated eRNA in T‐ALL.

These results suggest that BRD4 may influence the progression of T‐ALL by regulating HCP5.

### 3.3 BRD4 inhibitor significantly promotes HCP5 super‐enhancer activity and expression

BRD4 is an epigenetic regulator that modulates gene expression by binding to acetylated histones. It plays a crucial role in T‐ALL, primarily by regulating super‐enhancers and gene promoters to promote the expression of tumor‐related genes.^7^, ^17^ In the results described above, bioinformatic analysis identified HCP5, a lncRNA closely associated with BRD4, which has garnered increasing attention for its role in cancer in recent years.^32^

**FIGURE 3:**
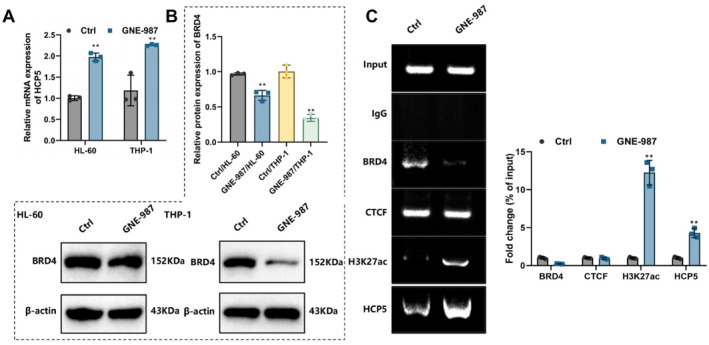
BRD4 inhibitor significantly promotes HCP5 super‐enhancer activity and expression. (A) Reverse transcription‐quantitative polymerase chain reaction analysis of HCP5 mRNA expression levels in acute myeloid leukemia cell lines (THP‐1 and HL‐60) treated with the BRD4 inhibitor GNE‐987. (B) Western Blot analysis showing BRD4 expression levels in different groups. (C) Representative agarose gel electrophoresis image and statistical quantification of ChIP‐qPCR products. Compared to the Ctrl group, *p < 0.05, **p < 0.01. All cell experiments were repeated three times. BRD4, Bromodomain‐containing protein 4; HCP5, HLA Complex P5.

To further explore the interaction between BRD4 and HCP5 and their roles in T‐ALL, we first conducted in vitro cell experiments to validate the effect of BRD4 regulation on the HCP5 super‐enhancer and its impact on HCP5 expression. First, AML cell lines THP‐1 and HL‐60 were cultured, and the cells were treated with a BRD4 inhibitor. Control and experimental groups were established. RT‐qPCR results showed a significant decrease in HCP5 mRNA expression levels in the experimental group (Figure 3A). Western blot analysis further confirmed a marked reduction in HCP5 protein levels (Figure 3B). These findings indicate that the BRD4 inhibitor GNE‐987 effectively promotes HCP5 gene expression.

Next, we used ChIP‐qPCR to detect BRD4 binding at the HCP5 super‐enhancer region. The results showed reduced BRD4 enrichment in the GNE‐987‐treated group, reflecting effective inhibition of BRD4 by the GNE‐987 inhibitor. HCP5 expression was significantly increased in the GNE‐987‐treated group, whereas it was nearly absent in the PBS group. The enrichment level of CTCF at the HCP5 super‐enhancer region remained relatively consistent between the two groups. CTCF is typically involved in maintaining the three‐dimensional structure of chromatin, and these results suggest that it is not directly affected by the BRD4 inhibitor.^33^ Additionally, H3K27ac and H3K4me1 levels were significantly elevated in the GNE‐987‐treated group (Figure 3C). H3K4me1 is a histone modification that marks enhancer regions and prepares them for transcriptional activation during differentiation.^34^, ^35^ Therefore, these results suggest increased super‐enhancer activity.

These findings indicate that BRD4 directly influences HCP5 gene expression by binding to the HCP5 super‐enhancer and regulating its activity.

### 3.4 BRD4 inhibitor GNE‐987 significantly inhibits AML cell proliferation and migration and induces apoptosis

**FIGURE 4:**
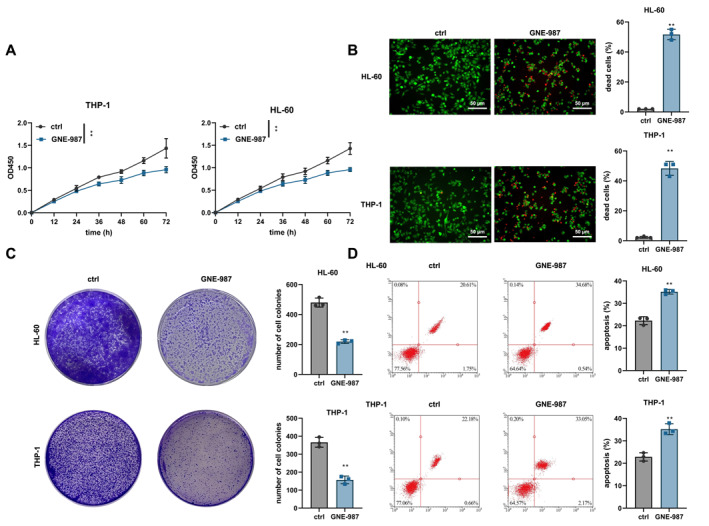
Bromodomain‐containing protein 4 Inhibitor GNE‐987 Significantly Inhibits acute myeloid leukemia Cell Proliferation and Migration and Induces Apoptosis. (A) CCK‐8 assay measuring cell proliferation in each group at 0, 12, 24, 36, 48, 60, and 72 h, with absorbance detected at OD450. (B) Representative images of Live and Dead staining for each group, with a bar chart depicting the statistical analysis of cell death ratios. Bar = 50 μm. (C) Colony formation assay for each group, with a bar chart showing the statistical analysis of colony numbers. (D) Flow cytometry analysis of apoptosis levels in each group, with a bar chart depicting the statistical analysis of apoptosis rates. Compared with the Ctrl group, *p < 0.05, **p < 0.01. All cell experiments were repeated three times.

AML cell lines (THP‐1 and HL‐60) were treated with the BRD4 inhibitor GNE‐987, and control and experimental groups were established to assess cell proliferation using the CCK‐8 assay. The results showed a significant reduction in cell proliferation in the GNE‐987‐treated group (Figure 4A). Live and dead cell staining and the statistical analysis of cell death and survival rates in each group revealed a significant increase in cell death in the GNE‐987‐treated cells, indicating that GNE‐987 effectively inhibited cell proliferation (Figure 4B). We further found that GNE‐987 negatively impacted colony formation, as the number of colonies in the GNE‐987 group was significantly reduced compared to the control group (Figure 4C). Flow cytometry analysis showed a significant increase in apoptosis in the GNE‐987‐treated group (Figure 4D).

**FIGURE 5:**
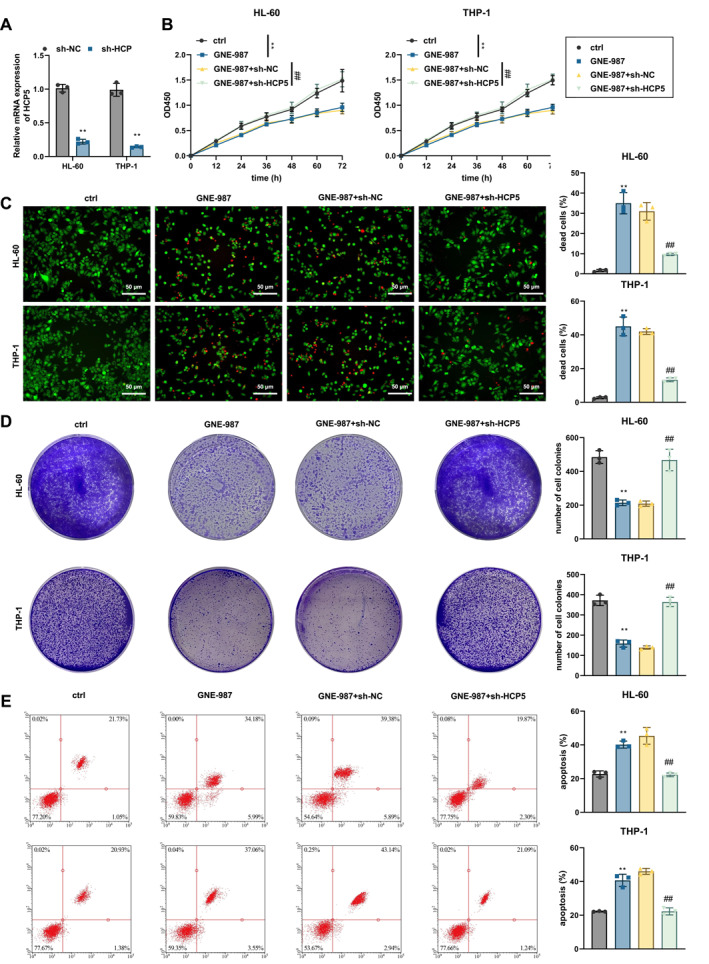
Silencing of HLA Complex P5 Reverses the Inhibitory Effect of GNE‐987 on acute myeloid leukemia Cell Viability. (A) Reverse transcription‐quantitative polymerase chain reaction results verify the silencing efficiency of sh‐HCP5. (B) CCK8 assay measuring cell proliferation at 0, 12, 24, 36, 48, 60, and 72 h, with absorbance detected at OD450. (C) Representative images of Live and Dead staining in each group and a bar chart showing the death ratio; scale bar = 50 μm. (D) Colony formation assay and statistical graph of colony numbers for each group. (E) Flow cytometry analysis of apoptosis levels and a statistical graph of apoptosis rates in each group. For panel A, compared with the sh‐NC group, *p < 0.05, **p < 0.01. For panels B‐E, compared with the Ctrl group, *p < 0.05, **p < 0.01; compared with the GNE‐987+sh‐NC group, #*p* < 0.05, ##*p* < 0.01. All cell experiments were repeated three times.

To investigate the critical role of HCP5 in the inhibitory effects of GNE‐987 on cell proliferation and migration, we silenced the HCP5 gene using lentivirus and further evaluated the effects of GNE‐987. RT‐qPCR confirmed the efficiency of HCP5 silencing (Figure 5A). The results indicated that silencing HCP5 reversed the inhibitory effects of GNE‐987 on cell viability (Figure 5B). Consistent results were also observed in T‐ALL–specific cell lines (Jurkat, CCRF‐CEM, MOLT‐4, and RPMI‐8402) (Figure S2). In the GNE‐987+sh‐HCP5 group, the cell viability inhibited by GNE‐987 was restored compared to the GNE‐987+sh‐NC group (Figure 5C). Similar results were observed in the colony formation assay, where silencing HCP5 reversed the inhibitory effect of GNE‐987 on colony formation (Figure 5D). Additionally, flow cytometry showed that sh‐HCP5 also reversed the pro‐apoptotic effect of GNE‐987 (Figure 5E).

These findings suggest that GNE‐987 effectively inhibits AML cell proliferation and migration and induces apoptosis by suppressing BRD4 binding to the HCP5 super‐enhancer, thereby reducing HCP5 expression.

### 3.5 BRD4 inhibitor GNE‐987 promotes HCP5 transcription and inhibits AML progression

**FIGURE 6:**
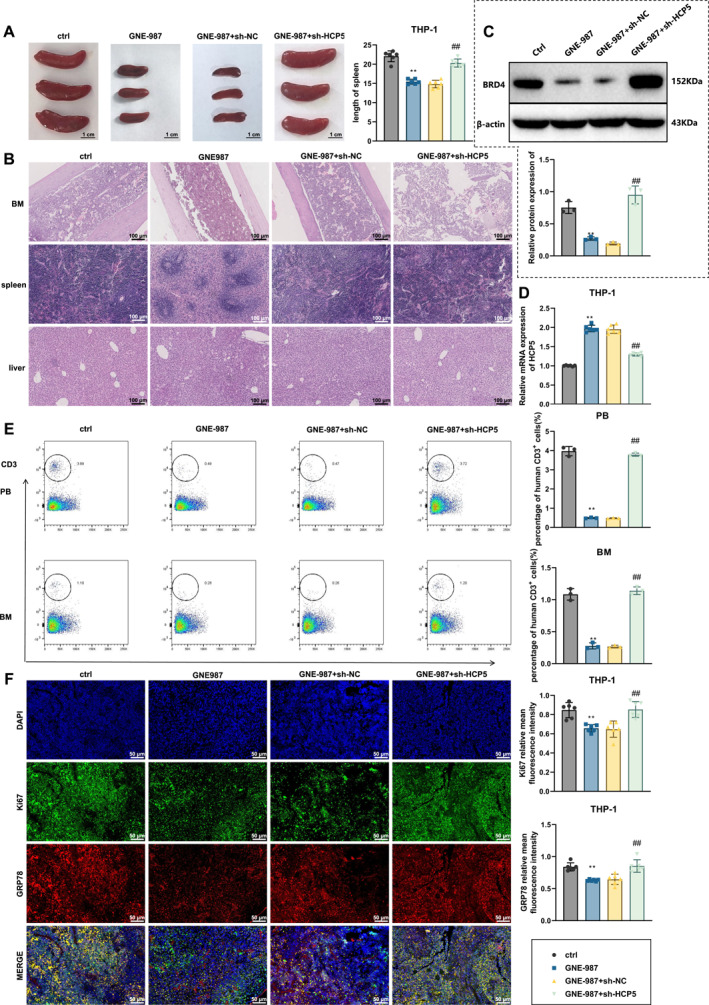
BRD4 Inhibitor GNE‐987 Promotes HCP5 Transcription and Inhibits acute myeloid leukemia Progression. (A) Gross appearance of mouse spleens and statistical graph of spleen length measurements, bar = 1 cm. (B) H&E staining of bone marrow, spleen, and liver tissues from each group of mice. (C) Western blot analysis of BRD4 protein expression in mouse spleen tissue and corresponding grayscale quantification; (D) reverse transcription‐quantitative polymerase chain reaction analysis of HCP5 mRNA levels in spleen tissue across groups; (E) flow cytometry analysis of human CD3+ cells in mice's peripheral blood and bone marrow. (F) Immunofluorescence staining of the mouse spleen for localization and quantification of Ki67 and GRP78; scale bar = 50 μm. Compared with the ctrl group, *p < 0.05, **p < 0.01; compared with the GNE‐987+sh‐NC group, #*p* < 0.05, ##*p* < 0.01. Each group included six mice. BRD4, Bromodomain‐containing protein 4; HCP5, HLA Complex P5.

By establishing an AML mouse model, HL‐60 cells were injected into BALB/c nude mice. Control and experimental groups were set up, with the experimental group receiving tail vein injections of the BRD4 inhibitor GNE‐987 (50 mg/kg), whereas the control group received an equivalent volume of PBS. Additionally, sh‐NC and sh‐HCP5 intervention groups were established, along with GNE‐987+sh‐NC and GNE‐987+sh‐HCP5 groups, to observe the role of HCP5 in the GNE‐987‐mediated effects. After 3 weeks, the mice were sacrificed to collect samples from peripheral blood, bone marrow, spleen, and liver. Compared with the control group, the spleens of mice in the GNE‐987 group were visibly smaller, whereas those in the GNE‐987+sh‐HCP5 group were larger. Spleen length was measured, and the results were consistent with the gross observations (Figure 6A).

We further evaluated organ infiltration through H&E staining. As shown in Figure 6B, compared to the control group, leukemic cell infiltration in the bone marrow, spleen, and liver of mice in the GNE‐987 group was significantly reduced, indicating that GNE‐987 exerts protective effects against T‐ALL in vivo. However, leukemic cell infiltration in mice's bone marrow, spleen, and liver in the GNE‐987+sh‐HCP5 group increased again, suggesting that sh‐HCP5 reversed the protective effects of GNE‐987.

We examined the expression of HCP5 and BRD4 in vivo. Results showed that BRD4 expression in the spleen of mice in the GNE‐987 group was significantly reduced, whereas HCP5 expression was significantly increased. The sh‐HCP5 intervention reversed the high HCP5 expression induced by GNE‐987 but had no effect on BRD4 expression (Figure 6C,D).

Consistent with the H&E staining results, flow cytometry showed that the percentage of hCD3+ cells in the peripheral blood and bone marrow of mice in the GNE‐987 group was significantly lower than that of the control group (Figure 6E).

Immunofluorescence staining showed that the nuclear expression of Ki67 and cytoplasmic expression of GRP78 in the spleen of mice in the GNE‐987 group were significantly lower than in the control group, whereas sh‐HCP5 upregulated their expression again (Figure 6F).

These results suggest that the BRD4 inhibitor GNE‐987 inhibits AML progression by targeting the HCP5 super‐enhancer, thereby downregulating HCP5 expression. These findings systematically validate the potential role of BRD4 in regulating HCP5 through super‐enhancers in treating AML.

## 4 DISCUSSION

BRD4, as a key member of the BET family, plays a critical regulatory role in various cancers.^36^ This study, through bioinformatics analysis and both in vivo and in vitro experiments, further clarifies the central role of BRD4 in T‐ALL. Previous studies have shown that BRD4 can promote the expression of tumor‐related genes and enhance the proliferation and survival of tumor cells by binding to super‐enhancers.^37^, ^38^ However, the specific mechanisms of BRD4 in T‐ALL, particularly its role in epigenetic regulation, still need to be understood.^39^ For the first time, this study links BRD4 with the HCP5 super‐enhancer, revealing BRD4's regulatory role in the proliferation of T‐ALL cells. Compared to previous studies, we confirmed the role of BRD4 in the proliferation and survival of T‐ALL cells and identified a novel molecular target, the HCP5 super‐enhancer. This finding complements existing research on BRD4 and super‐enhancer regulatory mechanisms, enriching our understanding of the pathogenesis of T‐ALL and providing a new perspective for BRD4‐targeted therapeutic strategies.

GNE‐987, a novel BRD4 inhibitor, has shown significant anti‐tumor activity in various cancers.^40^ This study is the first to validate the inhibitory effect of GNE‐987 in T‐ALL, particularly its ability to suppress cell proliferation and promote apoptosis by targeting the BRD4‐HCP5 regulatory axis. Previous studies on BRD4 inhibitors have predominantly focused on solid tumors, such as breast and prostate cancers, with limited application in hematological malignancies. In vitro and in vivo experiments demonstrated that GNE‐987 has unique therapeutic potential in T‐ALL, a specific type of hematologic malignancy. In this study, GNE‐987 demonstrated notable advantages in leukemia therapy. Existing BET inhibitors such as JQ1 and OTX015 exhibit certain anti‐proliferative effects in leukemia cells. For instance, JQ1 significantly inhibits proliferation and induces apoptosis in leukemia cell lines by competitively binding to the BRD4 bromodomain, thereby preventing its interaction with acetylated histones and suppressing tumor‐related gene expression. OTX015 has shown anti‐AML activity in preclinical studies and initial clinical efficacy.^41^ However, these inhibitors may cause hematopoietic toxicity and myelosuppression and are associated with acquired resistance during prolonged use. In contrast, GNE‐987 demonstrates unique advantages in potency, mechanism, and preclinical efficacy. It not only inhibits leukemia cell proliferation effectively but also suppresses BRD4 function through activation of the HCP5 super‐enhancer region, offering a novel therapeutic target. Furthermore, GNE‐987 significantly suppressed leukemia progression and improved pathology in mouse models, indicating strong clinical potential. These findings support further investigation and application of GNE‐987 in leukemia therapy.

Super‐enhancers have garnered widespread attention in cancer biology in recent years. As core elements regulate gene expression, super‐enhancers can significantly drive the overexpression of oncogenes.^42^ For example, oncogenes such as MYC and FOXO1 are driven by super‐enhancers in various malignancies, promoting rapid proliferation and malignant progression of tumor cells. This study is the first to reveal the role of the HCP5 super‐enhancer in T‐ALL, suggesting that it may be a key regulatory factor driving the proliferation of T‐ALL cells. Compared to super‐enhancers' role in other cancers, the specific regulation of HCP5 in T‐ALL provides a new target for tumor‐specific therapy. The cell‐specificity of super‐enhancers and their close association with specific oncogenes make targeting super‐enhancers a promising therapeutic strategy. This discovery further expands the scope of super‐enhancer research in hematological malignancies and provides a theoretical foundation for personalized treatment of T‐ALL.

Relapse and drug resistance remain major challenges in the clinical treatment of T‐ALL. Existing chemotherapies and targeted therapies often fail to completely control disease progression during long‐term treatment.^43^ Super‐enhancers and the oncogenes they regulate are considered key contributors to the development of drug resistance in tumor cells.^9^ BRD4, as a critical regulator of super‐enhancers, activates a range of tumor‐associated genes, enabling tumor cells to acquire enhanced survival capabilities and resistance to existing therapies.^44^, ^45^, ^46^ In this study, by inhibiting BRD4 and targeting the HCP5 super‐enhancer, we successfully blocked this regulatory pathway, significantly inhibiting the proliferation and survival of T‐ALL cells, thereby providing a new approach to overcoming drug resistance in T‐ALL. This finding is consistent with previous drug resistance studies, emphasizing the importance of BRD4 and super‐enhancers in tumor resistance and identifying new molecular targets in T‐ALL's resistance mechanisms.

To further validate the therapeutic potential of GNE‐987 in T‐ALL, we designed and conducted a series of in vitro cell experiments and in vivo animal studies. We confirmed that GNE‐987 effectively inhibits BRD4 expression and regulates T‐ALL cell bioactivity by targeting the HCP5 super‐enhancer through molecular biology techniques such as RT‐qPCR, Western blot, and ChIP‐qPCR. In animal experiments using a T‐ALL mouse model, we observed that GNE‐987 significantly inhibited tumor progression and improved mouse pathological manifestations. Compared to other BRD4 inhibitors, GNE‐987 exhibited more pronounced inhibitory effects, particularly in suppressing T‐ALL cell proliferation and promoting apoptosis. These findings provide crucial experimental evidence for the clinical application of GNE‐987 and offer valuable insights for future drug development and optimization.

One of the core findings of this study is the critical role of the HCP5 super‐enhancer in T‐ALL. Although the regulatory functions of super‐enhancers in cancer have been widely studied, the specific role of the HCP5 super‐enhancer in T‐ALL has not been thoroughly explored. Our experimental results demonstrate that the HCP5 super‐enhancer significantly influences T‐ALL cell proliferation and survival by regulating BRD4 expression. Targeting this super‐enhancer could provide a novel therapeutic strategy for T‐ALL, especially in the context of BRD4 inhibition by GNE‐987, with the regulation of the HCP5 super‐enhancer being a key component. Future studies could explore the potential of gene editing technologies or small‐molecule drugs to inhibit the HCP5 super‐enhancer, thereby offering more precise therapeutic options for T‐ALL patients.

Currently, molecular targeted therapies for T‐ALL primarily focus on regulating signaling pathways such as NOTCH1 and JAK/STAT. While these targeted drugs have shown promising effects in some patients, the heterogeneity of tumors and multiple drug resistance mechanisms often limit the long‐term efficacy of treatments targeting a single pathway. In contrast to these targets, BRD4 and its regulated super‐enhancer network offer another crucial tumor regulatory pathway. Our study found that GNE‐987, by modulating the BRD4‐HCP5 super‐enhancer axis, not only downregulates the expression of tumor‐related genes but also inhibits tumor cell proliferation, migration, and resistance through multiple mechanisms. The multi‐target characteristics of GNE‐987 may offer broader clinical applications, especially in combination therapies with other targeted drugs. GNE‐987 has the potential to enhance therapeutic efficacy and reduce the occurrence of drug resistance.

This study revealed the critical role of BRD4 in regulating the HCP5 gene through the super‐enhancer in AML progression and, for the first time, demonstrated the inhibitory effect of GNE‐987 on AML cells via the BRD4‐HCP5 axis. As a potent BRD4 inhibitor, GNE‐987 exhibits strong anti‐AML activity and holds promising potential for clinical application. This research offers a new target and therapeutic strategy for precision treatment of AML, especially for patients with high BRD4 expression and enhanced HCP5 activity, suggesting that GNE‐987 could become an effective treatment option.

Although this study demonstrates the therapeutic potential of GNE‐987 in AML, several limitations remain. First, the research was primarily conducted using cell lines and animal models, lacking clinical trial data; thus, further investigations are needed to evaluate its safety and efficacy in humans. Second, the regulatory mechanisms involving BRD4 and HCP5 may be more complex, potentially involving additional signaling pathways and molecular interactions, which require further elucidation. Moreover, long‐term use of GNE‐987 could lead to the development of drug resistance, highlighting the need for combination therapies to enhance treatment outcomes. Finally, the clinical applicability of GNE‐987 needs to be validated in larger‐scale clinical trials to ensure its efficacy and safety across diverse patient populations. Future studies should focus on advancing clinical trials to confirm the therapeutic value of GNE‐987, explore the broader regulatory networks of BRD4 and HCP5, and assess the potential of GNE‐987 in treating other subtypes of leukemia.

## 5 CONCLUSION

**FIGURE 7:**
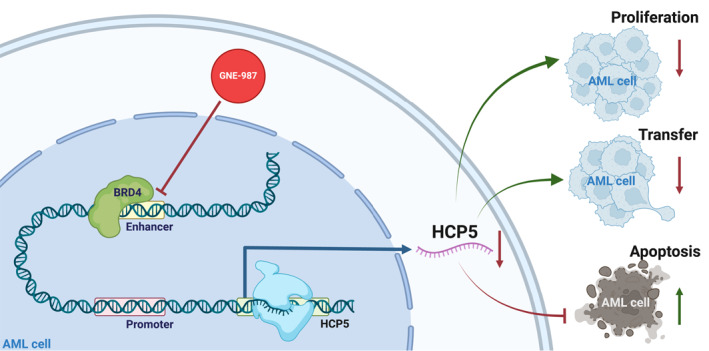
BRD4 inhibitor GNE‐987 activates the HLA Complex P5 super‐enhancer to downregulate BRD4 and inhibit T‐cell Acute Lymphoblastic Leukemia cell proliferation. BRD4, Bromodomain‐containing protein 4.

This study utilized multiple bioinformatics analyses and both in vitro and in vivo experiments to validate the potential mechanisms of the BRD4 inhibitor GNE‐987 in treating AML. The results demonstrated that BRD4 promotes AML cell proliferation and survival by binding to the HCP5 super‐enhancer and regulating its activity, which leads to the upregulation of HCP5 expression. GNE‐987 effectively inhibits BRD4 binding to the HCP5 super‐enhancer region, reduces HCP5 expression levels, significantly suppresses AML cell proliferation and migration, and induces apoptosis (mechanism depicted in Figure 7). In vivo, animal experiments further confirmed that GNE‐987 reduces AML cell infiltration in mice and significantly inhibits AML progression. These findings provide robust scientific evidence supporting the application of GNE‐987 in AML treatment.

## AUTHOR CONTRIBUTIONS

Xu Sang, Mengying Jiang, and Yanchun Guan contributed equally to this work as co‐first authors, performing the majority of experiments, data analysis, and manuscript drafting. Xin Chen provided technical assistance and experimental support. Zhen Zhang, Yumeng Wu, and Wansheng Peng conceived the study, supervised the experimental design, critically reviewed the data, and revised the manuscript for important intellectual content. All authors approved the final manuscript for submission.

## CONFLICT OF INTEREST STATEMENT

The authors declares no conflict of interest.

## ETHICS STATEMENT

All animal experiments were approved by the Experimental Animal Welfare Ethics Committee of The First Affiliated Hospital of Bengbu Medical University (approval number: 2024 No. 302).

## Supporting information

Supporting Information S1

Table S1

## Data Availability

All data can be provided as needed.
