# Supplementary material for: Mechanistic role of GNE‐987 targeting BRD4‐HCP5 axis in pediatric T‐cell acute lymphoblastic leukemia
Source: J Cell Commun Signal. 2026 Feb 14;20(1):e70063. doi: 10.1002/ccs3.70063 (PMC12906309; doi:10.1002/ccs3.70063)
Supplement: Supplementary file 1 — Supporting Information S1 [file CCS3-20-e70063-s002.docx]

**
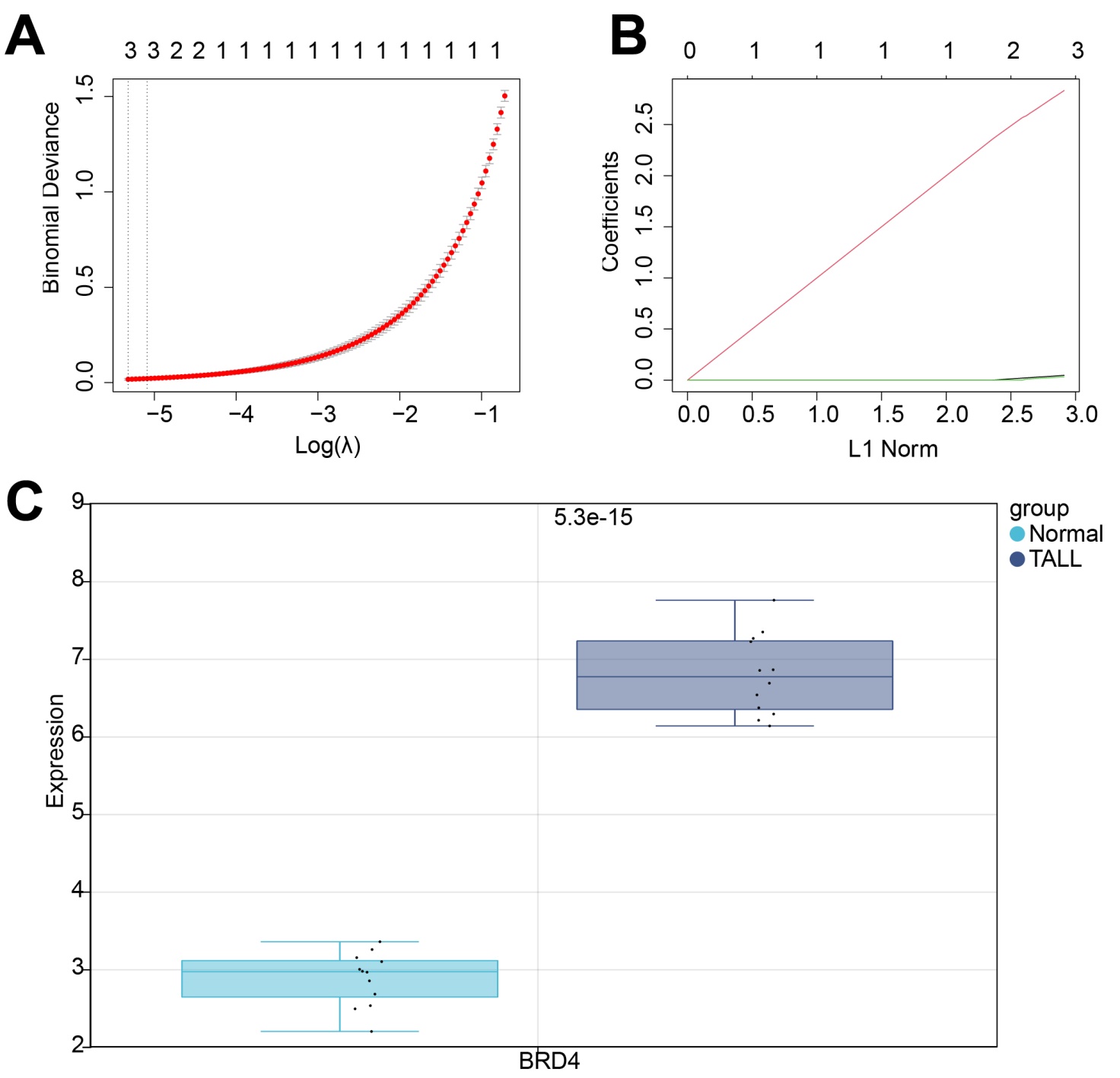
**

**Figure S1. LASSO Regression Analysis for Screening Key Factors.**

Note: (A) Distribution of LASSO coefficients for differentially expressed genes. (B) Selection of the optimal parameters (lambda) for the LASSO model. (C) Transcriptomic expression levels of BRD4. The sample size for each group is n = 12.

**
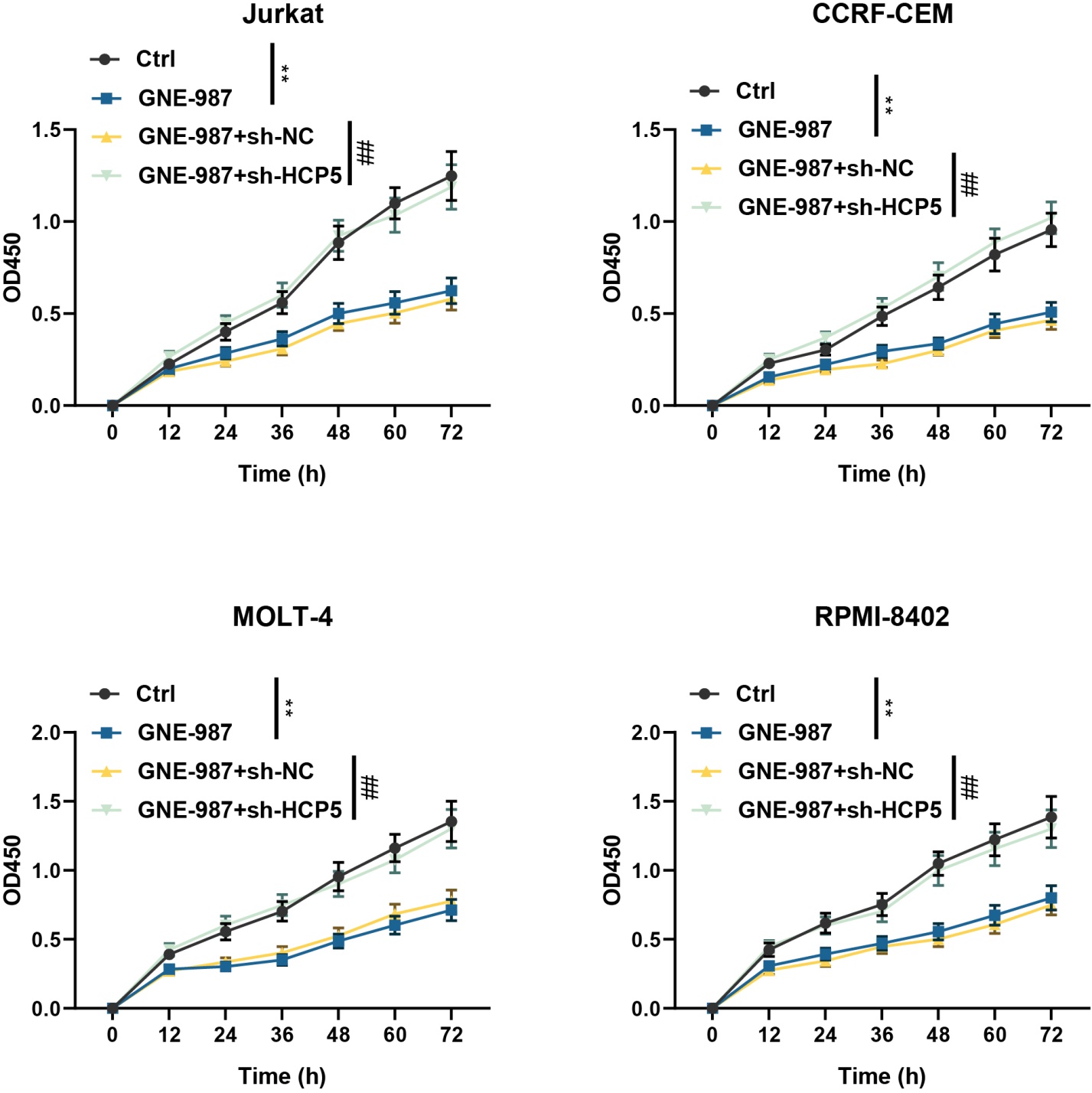
**

**Figure S2. HCP5 knockdown reverses GNE-987–induced proliferation inhibition in T-ALL cell lines.**

Note: CCK8 assays were performed to measure cell proliferation at 0, 12, 24, 36, 48, 60, and 72 hours, with absorbance measured at 450 nm. **p* < 0.05, ***p* < 0.01 vs. Ctrl group; #*p* < 0.05, ##*p* < 0.01 vs. GNE-987+sh-NC group. All experiments were performed in triplicate.
