# Supplementary material for: Mechanistic role of GNE‐987 targeting BRD4‐HCP5 axis in pediatric T‐cell acute lymphoblastic leukemia
Source: J Cell Commun Signal. 2026 Feb 14;20(1):e70063. doi: 10.1002/ccs3.70063 (PMC12906309; doi:10.1002/ccs3.70063)
Supplement: Supplementary file 2 — Table S1 [file CCS3-20-e70063-s001.docx]

Table S1. 791 genes closely related to BRD4.

| gene | conMean | treatMean | logFC | pValue | fdr |
| --- | --- | --- | --- | --- | --- |
| AADAT | 2.52840 | 5.30102 | 2.77263 | 0.000274392 | 0.01145 |
| AARS | 6.92550 | 10.08899 | 3.16349 | 7.17414E-05 | 0.00408 |
| ABCD3 | 6.98096 | 9.32028 | 2.33933 | 0.000102805 | 0.00545 |
| ACADM | 6.98798 | 10.23471 | 3.24673 | 0.000857939 | 0.02678 |
| ACAT2 | 5.31508 | 8.85550 | 3.54042 | 0.000656027 | 0.02163 |
| ACBD7 | 2.83218 | 6.54137 | 3.70920 | 2.21881E-05 | 0.00149 |
| ACOT7 | 4.65439 | 7.54234 | 2.88795 | 0.000495534 | 0.01752 |
| ADA | 6.55861 | 9.81613 | 3.25752 | 0.00037128 | 0.01423 |
| ADAM22 | 4.03415 | 7.13343 | 3.09929 | 0.001114581 | 0.03280 |
| ADAM9 | 3.84205 | 7.48272 | 3.64067 | 0.000857939 | 0.02678 |
| ADHFE1 | 6.37016 | 3.11862 | -3.25154 | 0.000274392 | 0.01145 |
| ADRBK2 | 3.64836 | 9.02999 | 5.38163 | 7.39602E-07 | 0.00010 |
| AFF2 | 3.86542 | 6.97422 | 3.10880 | 0.001114581 | 0.03280 |
| AGPAT5 | 6.17405 | 8.94667 | 2.77262 | 8.87523E-06 | 0.00071 |
| AHCY | 5.58730 | 8.54317 | 2.95587 | 1.4792E-06 | 0.00016 |
| AHNAK | 9.14256 | 5.20006 | -3.94250 | 7.39602E-07 | 0.00010 |
| AHR | 6.85541 | 2.59076 | -4.26465 | 5.17722E-06 | 0.00045 |
| AIG1 | 4.73599 | 7.43226 | 2.69627 | 0.00143261 | 0.04035 |
| AK5 | 6.03209 | 2.63503 | -3.39706 | 0.00037128 | 0.01423 |
| ALDH18A1 | 6.63109 | 9.44103 | 2.80994 | 7.39602E-07 | 0.00010 |
| ALDH1A2 | 3.24280 | 6.84858 | 3.60578 | 0.000857939 | 0.02678 |
| AMICA1 | 8.29547 | 3.86152 | -4.43395 | 5.17722E-06 | 0.00045 |
| AMY2A | 6.59093 | 4.66057 | -1.93036 | 0.001829776 | 0.04867 |
| ANKRD50 | 3.15886 | 7.09684 | 3.93798 | 8.87523E-06 | 0.00071 |
| ANLN | 2.82243 | 8.25759 | 5.43516 | 7.39602E-07 | 0.00010 |
| ANP32E | 6.48094 | 9.19781 | 2.71687 | 0.000656027 | 0.02163 |
| ANTXR1 | 2.81795 | 5.75974 | 2.94179 | 0.000274392 | 0.01145 |
| AOAH | 7.50390 | 4.44857 | -3.05533 | 0.000201172 | 0.00897 |
| ARHGAP11A | 3.60333 | 9.22205 | 5.61873 | 7.39602E-07 | 0.00010 |
| ARHGAP11B | 4.40184 | 8.45175 | 4.04991 | 7.17414E-05 | 0.00408 |
| ARHGAP19 | 5.85379 | 8.78183 | 2.92804 | 0.001829776 | 0.04867 |
| ARHGAP19-SLIT1 | 2.70663 | 5.54439 | 2.83776 | 0.000656027 | 0.02163 |
| ARL13B | 4.68444 | 7.64322 | 2.95878 | 0.001829776 | 0.04867 |
| ARMC1 | 5.87486 | 8.39340 | 2.51854 | 0.000274392 | 0.01145 |
| ARNTL2 | 2.91490 | 6.43411 | 3.51921 | 1.40524E-05 | 0.00103 |
| ARPP21 | 2.87842 | 6.87793 | 3.99952 | 0.000201172 | 0.00897 |
| ARRDC3 | 9.61431 | 6.09814 | -3.51617 | 0.001114581 | 0.03280 |
| ASF1B | 4.37313 | 9.02853 | 4.65540 | 7.39602E-07 | 0.00010 |
| ASPM | 2.84761 | 9.23294 | 6.38533 | 7.39602E-07 | 0.00010 |
| ASRGL1 | 3.65159 | 7.50223 | 3.85065 | 0.000144222 | 0.00719 |
| ATAD2 | 5.46507 | 9.43149 | 3.96642 | 2.21881E-05 | 0.00149 |
| ATAD5 | 4.42819 | 8.47454 | 4.04635 | 7.17414E-05 | 0.00408 |
| ATP5A1 | 8.63683 | 10.75146 | 2.11464 | 0.000656027 | 0.02163 |
| AUNIP | 3.02873 | 6.16686 | 3.13814 | 5.17722E-06 | 0.00045 |
| AURKA | 3.23076 | 8.40334 | 5.17258 | 7.39602E-07 | 0.00010 |
| AURKB | 4.82824 | 8.58173 | 3.75349 | 8.87523E-06 | 0.00071 |
| B4GALT6 | 3.14348 | 6.87399 | 3.73051 | 1.40524E-05 | 0.00103 |
| BARD1 | 4.57771 | 8.30350 | 3.72578 | 2.21881E-05 | 0.00149 |
| BCAT1 | 2.97775 | 8.72645 | 5.74870 | 7.39602E-07 | 0.00010 |
| BCL11A | 3.13067 | 6.52899 | 3.39832 | 0.000274392 | 0.01145 |
| BCL7A | 5.11945 | 7.89488 | 2.77544 | 0.00037128 | 0.01423 |
| BIRC5 | 3.23629 | 6.22207 | 2.98578 | 0.000144222 | 0.00719 |
| BLM | 4.61570 | 8.58012 | 3.96442 | 0.000201172 | 0.00897 |
| BRCA1 | 3.30753 | 7.94767 | 4.64015 | 7.39602E-07 | 0.00010 |
| BRCA2 | 3.37628 | 7.81085 | 4.43457 | 1.4792E-06 | 0.00016 |
| BRD4 | 2.87718 | 6.79191 | 3.91473 | 7.39602E-07 | 0.00010 |
| BRI3BP | 5.58196 | 8.46281 | 2.88085 | 0.001114581 | 0.03280 |
| BRIP1 | 3.73447 | 8.38917 | 4.65471 | 5.17722E-06 | 0.00045 |
| BTBD3 | 3.51228 | 7.55805 | 4.04578 | 0.000102805 | 0.00545 |
| BTG1 | 11.01469 | 8.23009 | -2.78460 | 1.40524E-05 | 0.00103 |
| BTG2 | 8.75535 | 6.21934 | -2.53601 | 0.00143261 | 0.04035 |
| BTLA | 6.56785 | 3.76368 | -2.80417 | 0.00037128 | 0.01423 |
| BUB1 | 3.54691 | 9.32810 | 5.78119 | 7.39602E-07 | 0.00010 |
| BUB1B | 3.17220 | 9.04104 | 5.86884 | 7.39602E-07 | 0.00010 |
| BYSL | 4.69495 | 8.24877 | 3.55383 | 7.39602E-07 | 0.00010 |
| C10orf25 | 3.28056 | 5.60112 | 2.32057 | 0.000857939 | 0.02678 |
| C18orf54 | 3.09353 | 6.34777 | 3.25424 | 0.000274392 | 0.01145 |
| C1orf112 | 3.76767 | 7.74029 | 3.97261 | 0.000144222 | 0.00719 |
| C1orf162 | 8.43889 | 4.69059 | -3.74831 | 2.21881E-05 | 0.00149 |
| C4orf46 | 4.76491 | 8.15406 | 3.38915 | 0.00143261 | 0.04035 |
| C5orf34 | 3.32853 | 6.61149 | 3.28295 | 0.000274392 | 0.01145 |
| C9orf47 | 3.45030 | 6.02496 | 2.57465 | 0.00037128 | 0.01423 |
| CA8 | 2.59109 | 5.61491 | 3.02382 | 0.000201172 | 0.00897 |
| CAPG | 5.01091 | 8.92524 | 3.91434 | 7.39602E-07 | 0.00010 |
| CAPN2 | 8.68749 | 4.67235 | -4.01514 | 2.95841E-06 | 0.00030 |
| CASC5 | 3.16773 | 8.96064 | 5.79290 | 7.39602E-07 | 0.00010 |
| CASP1 | 6.71777 | 3.20636 | -3.51140 | 0.000857939 | 0.02678 |
| CCDC138 | 3.52391 | 6.52094 | 2.99703 | 0.00037128 | 0.01423 |
| CCDC150 | 2.69115 | 5.45711 | 2.76596 | 0.000201172 | 0.00897 |
| CCDC34 | 3.46639 | 6.84633 | 3.37994 | 0.000274392 | 0.01145 |
| CCDC77 | 4.85147 | 7.61491 | 2.76344 | 0.00143261 | 0.04035 |
| CCDC88A | 4.50042 | 8.77470 | 4.27427 | 0.000201172 | 0.00897 |
| CCL5 | 9.50775 | 3.68767 | -5.82008 | 7.39602E-07 | 0.00010 |
| CCNA2 | 3.92164 | 9.14685 | 5.22521 | 7.39602E-07 | 0.00010 |
| CCNB1 | 3.57471 | 9.77565 | 6.20094 | 7.39602E-07 | 0.00010 |
| CCNB2 | 3.44103 | 9.45088 | 6.00984 | 7.39602E-07 | 0.00010 |
| CCND2 | 9.40463 | 5.25876 | -4.14587 | 0.000857939 | 0.02678 |
| CCNE2 | 3.64683 | 8.25837 | 4.61155 | 2.21881E-05 | 0.00149 |
| CCNF | 3.87316 | 7.62836 | 3.75520 | 7.39602E-07 | 0.00010 |
| CCR2 | 5.17431 | 2.92844 | -2.24587 | 0.000495534 | 0.01752 |
| CCR7 | 10.08895 | 6.04847 | -4.04048 | 7.17414E-05 | 0.00408 |
| CCR8 | 3.95501 | 6.83427 | 2.87925 | 0.000201172 | 0.00897 |
| CCT2 | 7.65614 | 10.31481 | 2.65868 | 1.40524E-05 | 0.00103 |
| CCT5 | 7.56532 | 9.88189 | 2.31658 | 4.95534E-05 | 0.00297 |
| CCT6A | 7.09339 | 9.81721 | 2.72382 | 2.95841E-06 | 0.00030 |
| CCT7 | 8.96150 | 10.74684 | 1.78534 | 0.000857939 | 0.02678 |
| CD1A | 3.03561 | 6.86495 | 3.82934 | 0.000495534 | 0.01752 |
| CD1B | 2.60908 | 6.65194 | 4.04286 | 0.000495534 | 0.01752 |
| CD1E | 3.45551 | 7.82480 | 4.36929 | 0.000656027 | 0.02163 |
| CD27 | 9.13276 | 5.01849 | -4.11427 | 2.95841E-06 | 0.00030 |
| CD37 | 9.90241 | 6.35957 | -3.54284 | 0.000102805 | 0.00545 |
| CD44 | 9.58630 | 5.75672 | -3.82957 | 0.001829776 | 0.04867 |
| CD55 | 8.40010 | 4.59018 | -3.80992 | 3.32821E-05 | 0.00210 |
| CD74 | 7.97546 | 4.39204 | -3.58342 | 2.21881E-05 | 0.00149 |
| CDC20 | 4.18110 | 8.46103 | 4.27993 | 1.4792E-06 | 0.00016 |
| CDC20P1 | 3.52560 | 5.86725 | 2.34166 | 0.00143261 | 0.04035 |
| CDC25A | 3.09377 | 7.97846 | 4.88469 | 7.39602E-07 | 0.00010 |
| CDC25C | 2.97760 | 6.93356 | 3.95596 | 1.4792E-06 | 0.00016 |
| CDC45 | 3.44015 | 8.58923 | 5.14908 | 7.39602E-07 | 0.00010 |
| CDC6 | 3.14917 | 9.28235 | 6.13318 | 7.39602E-07 | 0.00010 |
| CDC7 | 4.29883 | 8.14842 | 3.84959 | 2.21881E-05 | 0.00149 |
| CDCA2 | 2.92675 | 8.61656 | 5.68981 | 7.39602E-07 | 0.00010 |
| CDCA3 | 3.78333 | 7.35508 | 3.57175 | 2.95841E-06 | 0.00030 |
| CDCA5 | 3.44435 | 7.77166 | 4.32731 | 1.4792E-06 | 0.00016 |
| CDCA7 | 3.74679 | 9.04745 | 5.30066 | 7.39602E-07 | 0.00010 |
| CDCA7L | 5.84719 | 9.40841 | 3.56123 | 0.000201172 | 0.00897 |
| CDCA8 | 3.73043 | 7.40808 | 3.67765 | 8.87523E-06 | 0.00071 |
| CDH2 | 3.24353 | 6.84686 | 3.60332 | 0.00037128 | 0.01423 |
| CDK1 | 2.52564 | 8.37003 | 5.84438 | 7.39602E-07 | 0.00010 |
| CDK2 | 5.41198 | 9.47555 | 4.06357 | 7.17414E-05 | 0.00408 |
| CDK6 | 6.26804 | 9.93641 | 3.66836 | 0.00037128 | 0.01423 |
| CDKN2C | 3.50419 | 6.70066 | 3.19647 | 0.000656027 | 0.02163 |
| CDKN3 | 3.08799 | 7.73760 | 4.64960 | 2.95841E-06 | 0.00030 |
| CDT1 | 3.75011 | 7.86655 | 4.11644 | 5.17722E-06 | 0.00045 |
| CENPA | 3.86101 | 8.07397 | 4.21295 | 7.39602E-07 | 0.00010 |
| CENPE | 3.02816 | 6.96397 | 3.93581 | 2.21881E-05 | 0.00149 |
| CENPF | 3.74755 | 9.54483 | 5.79727 | 7.39602E-07 | 0.00010 |
| CENPI | 2.95748 | 8.48226 | 5.52478 | 7.39602E-07 | 0.00010 |
| CENPJ | 4.26589 | 7.89929 | 3.63340 | 0.000144222 | 0.00719 |
| CENPL | 3.98037 | 7.05359 | 3.07322 | 0.00037128 | 0.01423 |
| CENPM | 4.82778 | 8.32538 | 3.49760 | 3.32821E-05 | 0.00210 |
| CENPN | 4.03957 | 8.56998 | 4.53042 | 4.95534E-05 | 0.00297 |
| CENPO | 4.47350 | 8.36644 | 3.89294 | 0.000102805 | 0.00545 |
| CENPU | 3.38499 | 7.35054 | 3.96555 | 4.95534E-05 | 0.00297 |
| CENPW | 2.76978 | 7.55429 | 4.78451 | 7.39602E-07 | 0.00010 |
| CEP128 | 5.14154 | 8.81461 | 3.67307 | 0.00037128 | 0.01423 |
| CEP152 | 3.90674 | 7.41171 | 3.50497 | 0.00037128 | 0.01423 |
| CEP55 | 3.18291 | 8.43114 | 5.24824 | 7.39602E-07 | 0.00010 |
| CEP57L1 | 2.99038 | 6.30773 | 3.31734 | 0.000144222 | 0.00719 |
| CEP85 | 4.88016 | 8.08698 | 3.20682 | 0.000201172 | 0.00897 |
| CHAF1A | 5.06258 | 8.81007 | 3.74749 | 2.21881E-05 | 0.00149 |
| CHAF1B | 4.26347 | 8.34007 | 4.07660 | 8.87523E-06 | 0.00071 |
| CHCHD2 | 6.47722 | 9.75805 | 3.28083 | 0.000201172 | 0.00897 |
| CHD1L | 5.52868 | 8.13820 | 2.60952 | 0.000656027 | 0.02163 |
| CHEK1 | 4.18707 | 9.74416 | 5.55709 | 7.39602E-07 | 0.00010 |
| CHEK2 | 3.51365 | 6.10945 | 2.59580 | 0.00143261 | 0.04035 |
| CHRNA3 | 3.21216 | 6.01405 | 2.80190 | 0.000495534 | 0.01752 |
| CHRNA5 | 3.10537 | 7.51072 | 4.40536 | 7.39602E-07 | 0.00010 |
| CIT | 3.49058 | 8.44160 | 4.95102 | 1.4792E-06 | 0.00016 |
| CKAP2 | 5.75255 | 9.24560 | 3.49305 | 0.00037128 | 0.01423 |
| CKAP2L | 2.95349 | 7.38330 | 4.42980 | 7.39602E-07 | 0.00010 |
| CKAP5 | 6.60375 | 9.57754 | 2.97379 | 0.000495534 | 0.01752 |
| CKS1B | 4.57190 | 7.20804 | 2.63613 | 4.95534E-05 | 0.00297 |
| CKS2 | 4.35068 | 9.01340 | 4.66272 | 1.40524E-05 | 0.00103 |
| CLCN3 | 6.62460 | 8.38452 | 1.75991 | 0.000857939 | 0.02678 |
| CLIC4 | 3.97061 | 8.31015 | 4.33954 | 0.000857939 | 0.02678 |
| CLPTM1L | 7.32980 | 9.21380 | 1.88400 | 0.000495534 | 0.01752 |
| CLSPN | 2.95705 | 8.36230 | 5.40525 | 7.39602E-07 | 0.00010 |
| CNTLN | 2.65839 | 5.29467 | 2.63629 | 0.000495534 | 0.01752 |
| COA7 | 4.01790 | 6.82412 | 2.80622 | 0.001829776 | 0.04867 |
| CORO1C | 5.13252 | 9.56881 | 4.43629 | 7.39602E-07 | 0.00010 |
| CPS1 | 3.08602 | 5.34418 | 2.25816 | 0.001829776 | 0.04867 |
| CSGALNACT1 | 6.07025 | 3.16140 | -2.90885 | 0.000857939 | 0.02678 |
| CSNK1A1 | 8.15665 | 9.64856 | 1.49191 | 0.000656027 | 0.02163 |
| CTLA4 | 7.14789 | 3.09223 | -4.05566 | 1.4792E-06 | 0.00016 |
| CTNNAL1 | 3.06510 | 6.26390 | 3.19879 | 0.000274392 | 0.01145 |
| CTPS1 | 6.29367 | 9.44828 | 3.15461 | 8.87523E-06 | 0.00071 |
| CTSO | 5.88343 | 3.11969 | -2.76374 | 0.00143261 | 0.04035 |
| CTSS | 7.49316 | 4.49610 | -2.99705 | 0.000144222 | 0.00719 |
| CX3CR1 | 6.57841 | 3.30611 | -3.27230 | 0.001829776 | 0.04867 |
| CXorf24 | 4.09715 | 6.58758 | 2.49043 | 0.00037128 | 0.01423 |
| CYBRD1 | 3.61055 | 8.01928 | 4.40874 | 7.39602E-07 | 0.00010 |
| CYLD | 9.54143 | 6.64750 | -2.89393 | 7.17414E-05 | 0.00408 |
| CYSLTR1 | 5.90250 | 2.57544 | -3.32705 | 0.000201172 | 0.00897 |
| CYTH4 | 7.89103 | 5.51684 | -2.37420 | 0.000656027 | 0.02163 |
| CYTIP | 10.42650 | 3.93557 | -6.49093 | 7.39602E-07 | 0.00010 |
| DCBLD2 | 3.69934 | 6.57770 | 2.87836 | 0.001829776 | 0.04867 |
| DCLRE1A | 4.05167 | 6.89897 | 2.84730 | 0.001829776 | 0.04867 |
| DCUN1D5 | 5.34256 | 8.63581 | 3.29325 | 0.001114581 | 0.03280 |
| DDIAS | 3.11826 | 6.70420 | 3.58594 | 5.17722E-06 | 0.00045 |
| DDX39A | 6.40969 | 9.15624 | 2.74655 | 0.000201172 | 0.00897 |
| DDX50 | 7.53017 | 9.62994 | 2.09977 | 0.000274392 | 0.01145 |
| DEPDC1 | 2.33803 | 6.77242 | 4.43439 | 0.000201172 | 0.00897 |
| DEPDC1B | 3.19354 | 8.32409 | 5.13055 | 7.39602E-07 | 0.00010 |
| DHCR24 | 4.95508 | 8.59945 | 3.64437 | 0.001114581 | 0.03280 |
| DHFR | 5.41164 | 9.78801 | 4.37637 | 7.39602E-07 | 0.00010 |
| DHFRL1 | 5.02217 | 7.11191 | 2.08974 | 0.00143261 | 0.04035 |
| DHX15 | 8.00154 | 10.44287 | 2.44133 | 0.000274392 | 0.01145 |
| DIAPH3 | 2.74771 | 7.75104 | 5.00333 | 7.39602E-07 | 0.00010 |
| DLGAP5 | 2.82656 | 9.10400 | 6.27745 | 7.39602E-07 | 0.00010 |
| DMC1 | 2.78507 | 5.30972 | 2.52465 | 0.001114581 | 0.03280 |
| DNA2 | 3.76129 | 8.51821 | 4.75692 | 7.39602E-07 | 0.00010 |
| DNMT3B | 3.38340 | 6.31737 | 2.93396 | 0.000102805 | 0.00545 |
| DNTT | 2.98560 | 7.06109 | 4.07548 | 0.001114581 | 0.03280 |
| DOCK5 | 3.29399 | 6.07303 | 2.77904 | 0.001829776 | 0.04867 |
| DPP4 | 7.79002 | 3.72878 | -4.06125 | 0.000495534 | 0.01752 |
| DSC1 | 5.52435 | 2.75376 | -2.77058 | 0.000656027 | 0.02163 |
| DSCC1 | 2.84158 | 7.86160 | 5.02001 | 7.39602E-07 | 0.00010 |
| DTHD1 | 5.21948 | 2.64582 | -2.57366 | 0.00037128 | 0.01423 |
| DTL | 3.61304 | 9.75062 | 6.13759 | 7.39602E-07 | 0.00010 |
| DUSP1 | 9.56657 | 5.27429 | -4.29228 | 8.87523E-06 | 0.00071 |
| DUT | 5.56233 | 7.84291 | 2.28058 | 0.001114581 | 0.03280 |
| DYNLL1 | 6.48148 | 9.42134 | 2.93986 | 0.000857939 | 0.02678 |
| DYRK2 | 8.48955 | 6.53095 | -1.95861 | 0.000656027 | 0.02163 |
| DYRK3 | 3.14278 | 5.48164 | 2.33886 | 0.001114581 | 0.03280 |
| E2F2 | 3.84495 | 7.47411 | 3.62917 | 5.17722E-06 | 0.00045 |
| E2F7 | 3.39207 | 8.30596 | 4.91389 | 1.4792E-06 | 0.00016 |
| E2F8 | 3.22372 | 7.65258 | 4.42886 | 7.39602E-07 | 0.00010 |
| ECT2 | 3.45593 | 8.84370 | 5.38776 | 7.39602E-07 | 0.00010 |
| EIF4E3 | 8.43728 | 4.83666 | -3.60061 | 2.95841E-06 | 0.00030 |
| ELOVL4 | 3.69661 | 7.42512 | 3.72851 | 0.00143261 | 0.04035 |
| EME1 | 3.42685 | 7.01637 | 3.58951 | 0.000201172 | 0.00897 |
| ENO1 | 10.88056 | 12.32670 | 1.44614 | 3.32821E-05 | 0.00210 |
| EPDR1 | 3.67647 | 7.40939 | 3.73292 | 1.4792E-06 | 0.00016 |
| EPHA4 | 6.76430 | 3.78529 | -2.97901 | 4.95534E-05 | 0.00297 |
| ERCC6L | 2.94127 | 6.31082 | 3.36955 | 2.21881E-05 | 0.00149 |
| ESCO2 | 2.71995 | 7.79551 | 5.07556 | 7.39602E-07 | 0.00010 |
| ESPL1 | 3.43857 | 7.68644 | 4.24787 | 5.17722E-06 | 0.00045 |
| EXO1 | 3.24571 | 9.22742 | 5.98171 | 7.39602E-07 | 0.00010 |
| EXTL2 | 3.27489 | 6.20421 | 2.92932 | 0.000495534 | 0.01752 |
| EZH2 | 5.33878 | 9.44841 | 4.10963 | 2.21881E-05 | 0.00149 |
| FABP5 | 3.92922 | 8.64812 | 4.71890 | 7.39602E-07 | 0.00010 |
| FADS1 | 3.56024 | 8.34647 | 4.78623 | 8.87523E-06 | 0.00071 |
| FADS2 | 3.74566 | 9.23692 | 5.49126 | 5.17722E-06 | 0.00045 |
| FAIM3 | 8.91682 | 5.24984 | -3.66698 | 8.87523E-06 | 0.00071 |
| FAM173B | 4.71273 | 7.31954 | 2.60681 | 0.00037128 | 0.01423 |
| FAM27E2 | 3.52448 | 6.31934 | 2.79486 | 0.000857939 | 0.02678 |
| FAM64A | 3.71137 | 6.58963 | 2.87826 | 0.000495534 | 0.01752 |
| FAM65B | 9.54443 | 6.42755 | -3.11688 | 0.000144222 | 0.00719 |
| FAM72B | 3.55605 | 9.17378 | 5.61773 | 7.39602E-07 | 0.00010 |
| FANCA | 3.85518 | 7.75147 | 3.89629 | 2.21881E-05 | 0.00149 |
| FANCB | 3.49096 | 6.45705 | 2.96609 | 0.001114581 | 0.03280 |
| FANCD2 | 5.10924 | 8.69828 | 3.58903 | 0.000495534 | 0.01752 |
| FANCG | 4.50385 | 7.61364 | 3.10980 | 0.000495534 | 0.01752 |
| FANCI | 4.55902 | 10.20169 | 5.64267 | 7.39602E-07 | 0.00010 |
| FANCL | 3.89012 | 7.24597 | 3.35585 | 0.001114581 | 0.03280 |
| FASTKD1 | 5.16590 | 7.87630 | 2.71040 | 0.001829776 | 0.04867 |
| FAT1 | 3.17066 | 8.46055 | 5.28989 | 7.39602E-07 | 0.00010 |
| FBN1 | 2.90550 | 5.31598 | 2.41048 | 0.000495534 | 0.01752 |
| FBXO32 | 7.14669 | 3.92032 | -3.22636 | 0.000274392 | 0.01145 |
| FBXO43 | 2.69768 | 6.23484 | 3.53716 | 2.95841E-06 | 0.00030 |
| FBXO45 | 4.55175 | 7.74994 | 3.19819 | 0.000857939 | 0.02678 |
| FCRL6 | 7.18481 | 4.11907 | -3.06574 | 0.000656027 | 0.02163 |
| FDPS | 5.49720 | 8.64704 | 3.14984 | 0.000656027 | 0.02163 |
| FEN1 | 5.27685 | 8.99611 | 3.71925 | 7.39602E-07 | 0.00010 |
| FERMT2 | 2.75112 | 6.18004 | 3.42892 | 1.4792E-06 | 0.00016 |
| FGD6 | 2.96878 | 5.59308 | 2.62430 | 0.00143261 | 0.04035 |
| FH | 4.90549 | 7.75019 | 2.84470 | 0.000144222 | 0.00719 |
| FIGNL1 | 4.34592 | 8.02329 | 3.67737 | 0.000201172 | 0.00897 |
| FKBP3 | 5.20476 | 8.00755 | 2.80279 | 0.00037128 | 0.01423 |
| FKBP4 | 6.61466 | 8.92623 | 2.31157 | 0.001829776 | 0.04867 |
| FLJ36840 | 3.35593 | 8.11891 | 4.76298 | 1.4792E-06 | 0.00016 |
| FLT3LG | 9.10289 | 6.53425 | -2.56864 | 1.40524E-05 | 0.00103 |
| FNBP1L | 3.35448 | 6.49052 | 3.13603 | 0.000857939 | 0.02678 |
| FOS | 7.94577 | 4.83093 | -3.11484 | 0.001114581 | 0.03280 |
| FOXM1 | 3.40082 | 8.47768 | 5.07687 | 7.39602E-07 | 0.00010 |
| FRRS1 | 3.04240 | 5.85451 | 2.81212 | 0.000495534 | 0.01752 |
| GART | 6.14516 | 9.07301 | 2.92785 | 0.001829776 | 0.04867 |
| GBE1 | 4.89571 | 8.01712 | 3.12141 | 0.000495534 | 0.01752 |
| GBP2 | 7.90005 | 3.03216 | -4.86789 | 7.39602E-07 | 0.00010 |
| GBP4 | 7.52916 | 3.69720 | -3.83196 | 0.000144222 | 0.00719 |
| GBP5 | 8.50120 | 3.76819 | -4.73301 | 2.21881E-05 | 0.00149 |
| GEN1 | 4.24169 | 7.79259 | 3.55090 | 0.000274392 | 0.01145 |
| GGCT | 5.23982 | 7.83378 | 2.59396 | 0.000495534 | 0.01752 |
| GGH | 2.80827 | 6.28449 | 3.47622 | 0.00037128 | 0.01423 |
| GIMAP1 | 8.66822 | 5.97804 | -2.69018 | 0.000201172 | 0.00897 |
| GIMAP1-GIMAP5 | 9.47034 | 5.98876 | -3.48158 | 0.00037128 | 0.01423 |
| GIMAP4 | 8.64907 | 4.20603 | -4.44304 | 4.95534E-05 | 0.00297 |
| GIMAP7 | 9.09866 | 5.51442 | -3.58423 | 7.17414E-05 | 0.00408 |
| GIMAP8 | 6.53183 | 3.53074 | -3.00109 | 8.87523E-06 | 0.00071 |
| GINS1 | 3.32788 | 8.62479 | 5.29691 | 7.39602E-07 | 0.00010 |
| GINS2 | 3.92042 | 7.83428 | 3.91386 | 2.21881E-05 | 0.00149 |
| GINS3 | 3.73857 | 7.51165 | 3.77307 | 0.000144222 | 0.00719 |
| GINS4 | 4.12965 | 7.60599 | 3.47634 | 3.32821E-05 | 0.00210 |
| GJC1 | 3.47951 | 7.34121 | 3.86170 | 0.00037128 | 0.01423 |
| GLIPR1 | 8.61150 | 4.95015 | -3.66135 | 0.000857939 | 0.02678 |
| GLULP4 | 7.32317 | 10.51207 | 3.18891 | 0.000102805 | 0.00545 |
| GM2A | 5.87062 | 8.45677 | 2.58615 | 0.001829776 | 0.04867 |
| GMNN | 3.74601 | 7.95352 | 4.20751 | 5.17722E-06 | 0.00045 |
| GNA15 | 3.98485 | 8.50478 | 4.51994 | 3.32821E-05 | 0.00210 |
| GPR125 | 3.78506 | 7.12413 | 3.33907 | 0.000102805 | 0.00545 |
| GPR137C | 2.95175 | 5.43768 | 2.48593 | 0.000495534 | 0.01752 |
| GPR15 | 7.26396 | 3.00282 | -4.26114 | 0.000274392 | 0.01145 |
| GPR171 | 6.56889 | 3.51899 | -3.04990 | 0.001829776 | 0.04867 |
| GPRIN3 | 7.96282 | 4.08468 | -3.87814 | 7.39602E-07 | 0.00010 |
| GPSM2 | 3.42177 | 8.39659 | 4.97481 | 7.39602E-07 | 0.00010 |
| GPT2 | 3.48612 | 6.54469 | 3.05856 | 0.00143261 | 0.04035 |
| GSAP | 6.07361 | 2.93422 | -3.13939 | 0.00143261 | 0.04035 |
| GSG2 | 3.44682 | 6.88072 | 3.43390 | 2.21881E-05 | 0.00149 |
| GSTCD | 3.90419 | 6.93940 | 3.03521 | 0.001829776 | 0.04867 |
| GTPBP4 | 6.38772 | 8.71333 | 2.32560 | 7.17414E-05 | 0.00408 |
| GTSE1 | 3.39941 | 8.12678 | 4.72737 | 7.39602E-07 | 0.00010 |
| GXYLT2 | 3.08678 | 7.75510 | 4.66833 | 0.00143261 | 0.04035 |
| GZMK | 8.62103 | 2.47947 | -6.14156 | 7.39602E-07 | 0.00010 |
| H2AFZ | 7.35551 | 11.08214 | 3.72663 | 4.95534E-05 | 0.00297 |
| HADH | 5.65933 | 8.95533 | 3.29600 | 0.000495534 | 0.01752 |
| HCP5 | 8.21661 | 4.01187 | -4.20473 | 2.95841E-06 | 0.00030 |
| HCST | 8.95633 | 7.18224 | -1.77410 | 0.00143261 | 0.04035 |
| HDGFRP3 | 4.42387 | 8.12988 | 3.70600 | 0.000274392 | 0.01145 |
| HELLS | 3.66743 | 8.82830 | 5.16087 | 7.39602E-07 | 0.00010 |
| HENMT1 | 4.95190 | 7.86542 | 2.91352 | 1.40524E-05 | 0.00103 |
| HHIP | 2.99401 | 7.78805 | 4.79404 | 7.17414E-05 | 0.00408 |
| HILPDA | 3.62195 | 7.08608 | 3.46413 | 0.000274392 | 0.01145 |
| HIRIP3 | 4.44659 | 7.19374 | 2.74715 | 0.000274392 | 0.01145 |
| HIST1H1B | 5.38185 | 11.01834 | 5.63648 | 7.39602E-07 | 0.00010 |
| HIST1H1D | 6.63976 | 9.67510 | 3.03534 | 0.000102805 | 0.00545 |
| HIST1H2AB | 5.20687 | 9.81282 | 4.60595 | 7.39602E-07 | 0.00010 |
| HIST1H2AG | 5.23554 | 8.12662 | 2.89108 | 0.00037128 | 0.01423 |
| HIST1H2AJ | 2.71916 | 5.10373 | 2.38458 | 0.001114581 | 0.03280 |
| HIST1H2AL | 3.90017 | 7.11751 | 3.21734 | 0.00143261 | 0.04035 |
| HIST1H2BC | 4.22996 | 8.01311 | 3.78314 | 0.000656027 | 0.02163 |
| HIST1H2BJ | 3.31669 | 5.95148 | 2.63479 | 0.001114581 | 0.03280 |
| HIST1H2BO | 3.88563 | 6.84391 | 2.95828 | 0.001114581 | 0.03280 |
| HIST1H3B | 5.01186 | 11.10908 | 6.09722 | 7.39602E-07 | 0.00010 |
| HIST1H3F | 5.19308 | 8.84521 | 3.65213 | 0.000857939 | 0.02678 |
| HIST1H3G | 3.57220 | 7.83912 | 4.26692 | 0.000201172 | 0.00897 |
| HIST1H3H | 5.18865 | 8.80128 | 3.61263 | 0.001829776 | 0.04867 |
| HIST1H3I | 8.41738 | 12.16697 | 3.74959 | 7.17414E-05 | 0.00408 |
| HIST1H3J | 4.06026 | 8.63963 | 4.57937 | 5.17722E-06 | 0.00045 |
| HIST1H4A | 5.25913 | 8.81436 | 3.55523 | 3.32821E-05 | 0.00210 |
| HIST1H4B | 6.86790 | 10.38729 | 3.51939 | 7.17414E-05 | 0.00408 |
| HIST1H4D | 4.92594 | 9.15262 | 4.22668 | 0.00037128 | 0.01423 |
| HIST1H4K | 4.47678 | 6.41036 | 1.93358 | 0.000495534 | 0.01752 |
| HIST1H4L | 5.29370 | 9.95882 | 4.66512 | 1.4792E-06 | 0.00016 |
| HIST2H2AA4 | 7.60788 | 11.28720 | 3.67932 | 8.87523E-06 | 0.00071 |
| HIST2H2AB | 7.99497 | 11.93142 | 3.93645 | 5.17722E-06 | 0.00045 |
| HIST2H3A | 5.95947 | 9.89016 | 3.93069 | 0.000656027 | 0.02163 |
| HJURP | 3.55456 | 8.46749 | 4.91293 | 7.39602E-07 | 0.00010 |
| HLA-DPB1 | 6.81955 | 3.50446 | -3.31509 | 0.000102805 | 0.00545 |
| HLA-F | 9.05321 | 3.91551 | -5.13769 | 7.39602E-07 | 0.00010 |
| HLTF | 5.32077 | 8.72381 | 3.40304 | 0.000656027 | 0.02163 |
| HMGB1P10 | 6.62383 | 9.61366 | 2.98984 | 8.87523E-06 | 0.00071 |
| HMGB2 | 6.18703 | 9.89840 | 3.71137 | 4.95534E-05 | 0.00297 |
| HMGB3 | 3.75706 | 7.04431 | 3.28724 | 0.000274392 | 0.01145 |
| HMGCR | 5.81096 | 9.54878 | 3.73781 | 0.000144222 | 0.00719 |
| HMGCS1 | 5.37851 | 9.27121 | 3.89270 | 0.00037128 | 0.01423 |
| HMGN5 | 3.35600 | 6.32145 | 2.96546 | 0.000656027 | 0.02163 |
| HMMR | 2.78105 | 7.59405 | 4.81301 | 7.39602E-07 | 0.00010 |
| HNRNPA1P12 | 7.43114 | 10.14081 | 2.70967 | 2.95841E-06 | 0.00030 |
| HNRNPAB | 8.20443 | 10.71732 | 2.51288 | 0.000495534 | 0.01752 |
| HNRNPF | 7.85992 | 10.22689 | 2.36697 | 1.40524E-05 | 0.00103 |
| HNRNPH1 | 10.08041 | 11.31890 | 1.23849 | 0.000656027 | 0.02163 |
| HNRNPM | 9.15592 | 10.52189 | 1.36597 | 0.000656027 | 0.02163 |
| HNRNPR | 8.45689 | 10.39716 | 1.94027 | 0.001114581 | 0.03280 |
| HOOK1 | 4.75242 | 2.49454 | -2.25788 | 0.000656027 | 0.02163 |
| HS2ST1 | 6.17287 | 8.48997 | 2.31709 | 0.000495534 | 0.01752 |
| HSD17B6 | 2.62314 | 4.56465 | 1.94150 | 0.001114581 | 0.03280 |
| HSPA4L | 2.39756 | 5.83501 | 3.43745 | 0.000201172 | 0.00897 |
| HSPD1 | 7.64735 | 10.52982 | 2.88247 | 2.21881E-05 | 0.00149 |
| IARS | 8.35045 | 10.78536 | 2.43492 | 0.000201172 | 0.00897 |
| ICOS | 7.51449 | 3.54384 | -3.97065 | 0.000495534 | 0.01752 |
| IDH1 | 5.28167 | 8.37661 | 3.09495 | 0.000495534 | 0.01752 |
| IDH2 | 7.38503 | 10.05971 | 2.67468 | 0.00037128 | 0.01423 |
| IFI44L | 6.53074 | 3.13789 | -3.39285 | 0.000857939 | 0.02678 |
| IFT81 | 2.76327 | 4.68698 | 1.92371 | 0.000857939 | 0.02678 |
| IGFBP2 | 4.35445 | 8.14074 | 3.78629 | 0.000201172 | 0.00897 |
| IKZF3 | 8.65695 | 5.11437 | -3.54258 | 0.000102805 | 0.00545 |
| IL10RA | 9.33823 | 4.32236 | -5.01588 | 7.39602E-07 | 0.00010 |
| IL2RB | 7.71146 | 4.93558 | -2.77587 | 1.40524E-05 | 0.00103 |
| IL6R | 7.65921 | 4.13016 | -3.52905 | 1.4792E-06 | 0.00016 |
| IL6ST | 8.92511 | 5.04417 | -3.88095 | 7.17414E-05 | 0.00408 |
| IL7R | 10.94647 | 4.82370 | -6.12277 | 7.39602E-07 | 0.00010 |
| IMPDH2 | 7.44830 | 9.88814 | 2.43984 | 0.001829776 | 0.04867 |
| INA | 3.01416 | 5.22690 | 2.21274 | 0.001829776 | 0.04867 |
| INADL | 6.70037 | 2.98154 | -3.71883 | 1.4792E-06 | 0.00016 |
| INPP4B | 8.67853 | 3.95927 | -4.71926 | 8.87523E-06 | 0.00071 |
| INSIG1 | 6.53646 | 9.71007 | 3.17360 | 0.00037128 | 0.01423 |
| INTS2 | 6.12317 | 8.53659 | 2.41342 | 0.00143261 | 0.04035 |
| INTS7 | 5.39326 | 8.71206 | 3.31880 | 0.001829776 | 0.04867 |
| IPCEF1 | 8.90468 | 5.36706 | -3.53762 | 4.95534E-05 | 0.00297 |
| IQGAP3 | 3.21640 | 5.99284 | 2.77644 | 0.000857939 | 0.02678 |
| ITGB7 | 8.85191 | 5.83413 | -3.01778 | 0.000144222 | 0.00719 |
| JADE3 | 3.05390 | 6.77249 | 3.71859 | 0.000201172 | 0.00897 |
| JAK3 | 7.85559 | 5.58307 | -2.27252 | 0.001114581 | 0.03280 |
| JPH1 | 3.21471 | 7.01510 | 3.80040 | 7.39602E-07 | 0.00010 |
| JRKL | 5.17202 | 7.67025 | 2.49823 | 0.000656027 | 0.02163 |
| JUNB | 9.27934 | 5.95245 | -3.32689 | 7.17414E-05 | 0.00408 |
| KCNK5 | 3.37256 | 6.36278 | 2.99022 | 1.40524E-05 | 0.00103 |
| KCTD3 | 3.48497 | 6.55488 | 3.06991 | 0.00037128 | 0.01423 |
| KDELC1 | 2.94239 | 5.09395 | 2.15156 | 0.00143261 | 0.04035 |
| KDM1A | 6.93057 | 9.36586 | 2.43530 | 0.000495534 | 0.01752 |
| KIAA0101 | 3.18253 | 9.00547 | 5.82295 | 7.39602E-07 | 0.00010 |
| KIAA1524 | 3.96336 | 8.70989 | 4.74652 | 7.39602E-07 | 0.00010 |
| KIF11 | 3.39451 | 8.82294 | 5.42843 | 7.39602E-07 | 0.00010 |
| KIF14 | 2.65173 | 8.12022 | 5.46849 | 7.39602E-07 | 0.00010 |
| KIF15 | 2.78759 | 8.04866 | 5.26107 | 7.39602E-07 | 0.00010 |
| KIF18A | 3.21375 | 7.58509 | 4.37134 | 2.95841E-06 | 0.00030 |
| KIF18B | 3.73233 | 7.08150 | 3.34917 | 3.32821E-05 | 0.00210 |
| KIF20A | 2.69854 | 8.10235 | 5.40380 | 7.39602E-07 | 0.00010 |
| KIF23 | 3.24673 | 9.27410 | 6.02737 | 7.39602E-07 | 0.00010 |
| KIF24 | 3.03220 | 5.79540 | 2.76320 | 0.000144222 | 0.00719 |
| KIF2C | 3.29453 | 8.64692 | 5.35239 | 7.39602E-07 | 0.00010 |
| KIF4A | 2.95602 | 8.39299 | 5.43697 | 7.39602E-07 | 0.00010 |
| KIF4B | 2.86106 | 6.28870 | 3.42763 | 5.17722E-06 | 0.00045 |
| KIFC1 | 3.27147 | 6.28281 | 3.01134 | 5.17722E-06 | 0.00045 |
| KLF2 | 9.50751 | 6.46175 | -3.04575 | 7.39602E-07 | 0.00010 |
| KLF3 | 8.53292 | 3.70349 | -4.82942 | 7.39602E-07 | 0.00010 |
| KLF9 | 7.43224 | 4.74193 | -2.69030 | 1.4792E-06 | 0.00016 |
| KLRB1 | 7.12282 | 2.95781 | -4.16501 | 3.32821E-05 | 0.00210 |
| KLRC4 | 5.95340 | 2.56441 | -3.38899 | 0.001114581 | 0.03280 |
| KLRC4-KLRK1 | 7.25725 | 3.25106 | -4.00619 | 0.000495534 | 0.01752 |
| KLRG1 | 8.58374 | 4.80238 | -3.78137 | 8.87523E-06 | 0.00071 |
| KNSTRN | 4.49363 | 8.78041 | 4.28678 | 3.32821E-05 | 0.00210 |
| KNTC1 | 4.87260 | 8.83869 | 3.96609 | 0.000201172 | 0.00897 |
| KPNA2 | 6.53151 | 10.76467 | 4.23316 | 7.17414E-05 | 0.00408 |
| LBH | 8.60307 | 6.26461 | -2.33846 | 3.32821E-05 | 0.00210 |
| LBR | 8.87419 | 10.88075 | 2.00657 | 0.000144222 | 0.00719 |
| LDLRAP1 | 8.85940 | 4.83620 | -4.02320 | 7.39602E-07 | 0.00010 |
| LINC00909 | 5.15891 | 7.37718 | 2.21827 | 0.001829776 | 0.04867 |
| LINC01366 | 3.12022 | 5.74799 | 2.62777 | 0.000495534 | 0.01752 |
| LITAF | 9.27389 | 5.77228 | -3.50161 | 0.000201172 | 0.00897 |
| LMNB1 | 5.27115 | 9.88646 | 4.61531 | 7.39602E-07 | 0.00010 |
| LOC100131541 | 7.22323 | 3.53985 | -3.68338 | 4.95534E-05 | 0.00297 |
| LOC100288637 | 3.34831 | 6.56728 | 3.21897 | 0.000274392 | 0.01145 |
| LOC101060546 | 3.45945 | 6.14962 | 2.69017 | 0.001829776 | 0.04867 |
| LONP1 | 5.44942 | 7.79041 | 2.34099 | 0.001829776 | 0.04867 |
| LRP12 | 3.16461 | 7.60224 | 4.43763 | 7.39602E-07 | 0.00010 |
| LRRC1 | 3.85511 | 6.44508 | 2.58997 | 0.001114581 | 0.03280 |
| LRRC34 | 2.64753 | 5.48780 | 2.84027 | 5.17722E-06 | 0.00045 |
| LRRN3 | 7.62096 | 3.12178 | -4.49918 | 2.95841E-06 | 0.00030 |
| LTBP1 | 3.06215 | 5.90608 | 2.84393 | 7.17414E-05 | 0.00408 |
| MAD2L1 | 4.53514 | 8.86965 | 4.33451 | 7.39602E-07 | 0.00010 |
| MAML2 | 8.20545 | 3.28981 | -4.91564 | 7.39602E-07 | 0.00010 |
| MAP1A | 3.49169 | 6.72072 | 3.22903 | 0.000201172 | 0.00897 |
| MAP3K1 | 9.34425 | 6.92104 | -2.42321 | 0.000102805 | 0.00545 |
| MCM10 | 3.26416 | 8.84926 | 5.58509 | 7.39602E-07 | 0.00010 |
| MCM2 | 4.55151 | 8.85487 | 4.30336 | 7.39602E-07 | 0.00010 |
| MCM3 | 6.55469 | 9.71597 | 3.16128 | 0.000102805 | 0.00545 |
| MCM4 | 4.66490 | 9.11776 | 4.45286 | 7.39602E-07 | 0.00010 |
| MCM5 | 6.49437 | 9.49102 | 2.99665 | 2.21881E-05 | 0.00149 |
| MCM6 | 6.29248 | 10.23315 | 3.94067 | 7.39602E-07 | 0.00010 |
| MCM7 | 7.24732 | 10.36064 | 3.11332 | 5.17722E-06 | 0.00045 |
| MED12L | 2.95759 | 7.87885 | 4.92126 | 7.39602E-07 | 0.00010 |
| MED20 | 4.89187 | 7.52192 | 2.63005 | 0.000656027 | 0.02163 |
| MELK | 3.09388 | 9.09160 | 5.99773 | 7.39602E-07 | 0.00010 |
| MFAP1 | 4.86662 | 7.57946 | 2.71283 | 0.001829776 | 0.04867 |
| MGC40069 | 5.94720 | 2.95829 | -2.98891 | 0.00037128 | 0.01423 |
| MGME1 | 4.81354 | 7.97636 | 3.16282 | 0.000201172 | 0.00897 |
| MID1 | 2.55163 | 4.35390 | 1.80227 | 0.001114581 | 0.03280 |
| MID2 | 6.07054 | 2.93258 | -3.13796 | 0.000274392 | 0.01145 |
| MINOS1 | 7.79691 | 9.35270 | 1.55579 | 7.17414E-05 | 0.00408 |
| MIR15A | 3.72939 | 7.50301 | 3.77362 | 0.001114581 | 0.03280 |
| MIS18A | 5.34842 | 8.97132 | 3.62290 | 7.39602E-07 | 0.00010 |
| MKI67 | 3.95200 | 9.88678 | 5.93478 | 7.39602E-07 | 0.00010 |
| MKRN3 | 4.36238 | 7.23897 | 2.87659 | 0.000201172 | 0.00897 |
| MLEC | 5.94685 | 8.63157 | 2.68472 | 0.000656027 | 0.02163 |
| MMS22L | 4.57003 | 8.31521 | 3.74518 | 0.000201172 | 0.00897 |
| MND1 | 2.90615 | 7.76127 | 4.85512 | 7.39602E-07 | 0.00010 |
| MNS1 | 2.50538 | 4.99061 | 2.48523 | 0.000857939 | 0.02678 |
| MPHOSPH6 | 5.14197 | 7.95665 | 2.81468 | 0.001114581 | 0.03280 |
| MPZL1 | 4.20623 | 7.90338 | 3.69715 | 8.87523E-06 | 0.00071 |
| MREG | 4.10310 | 6.76926 | 2.66616 | 0.000201172 | 0.00897 |
| MRPL2 | 6.49709 | 8.23228 | 1.73518 | 0.001829776 | 0.04867 |
| MSANTD3-TMEFF1 | 3.68446 | 6.61514 | 2.93068 | 0.00143261 | 0.04035 |
| MSH5 | 4.66381 | 8.35062 | 3.68680 | 0.000201172 | 0.00897 |
| MSH5-SAPCD1 | 4.12417 | 7.42961 | 3.30545 | 0.000201172 | 0.00897 |
| MT-TE | 8.89708 | 11.70080 | 2.80372 | 7.39602E-07 | 0.00010 |
| MT-TH | 8.32673 | 11.18896 | 2.86223 | 0.000274392 | 0.01145 |
| MT-TS1 | 8.80543 | 10.62049 | 1.81507 | 2.95841E-06 | 0.00030 |
| MT1F | 8.18360 | 5.88896 | -2.29465 | 0.001829776 | 0.04867 |
| MTCH2 | 7.18326 | 9.87555 | 2.69229 | 0.001114581 | 0.03280 |
| MTF2 | 7.75511 | 10.03407 | 2.27896 | 0.000656027 | 0.02163 |
| MTFR2 | 3.46366 | 7.19073 | 3.72707 | 1.40524E-05 | 0.00103 |
| MTHFD1 | 6.13019 | 9.54523 | 3.41503 | 1.4792E-06 | 0.00016 |
| MTHFD1L | 5.07713 | 8.27991 | 3.20277 | 0.00037128 | 0.01423 |
| MTHFD2 | 5.76539 | 10.64665 | 4.88126 | 7.39602E-07 | 0.00010 |
| MTHFD2P7 | 4.56744 | 9.90838 | 5.34094 | 7.39602E-07 | 0.00010 |
| MVP | 6.87339 | 4.88841 | -1.98499 | 4.95534E-05 | 0.00297 |
| MYB | 5.47546 | 10.36081 | 4.88535 | 3.32821E-05 | 0.00210 |
| MYBL2 | 4.12287 | 9.15863 | 5.03576 | 7.39602E-07 | 0.00010 |
| MYH10 | 3.51736 | 8.11730 | 4.59993 | 0.000201172 | 0.00897 |
| MYL6B | 4.68214 | 7.18801 | 2.50587 | 0.000495534 | 0.01752 |
| MYLIP | 8.51719 | 5.02387 | -3.49332 | 2.21881E-05 | 0.00149 |
| MZB1 | 3.80312 | 7.99872 | 4.19560 | 1.4792E-06 | 0.00016 |
| N6AMT2 | 4.30587 | 6.95649 | 2.65062 | 0.000495534 | 0.01752 |
| NASP | 5.83406 | 9.13590 | 3.30184 | 7.17414E-05 | 0.00408 |
| NCAPD3 | 6.28982 | 9.59672 | 3.30690 | 0.000201172 | 0.00897 |
| NCAPG | 3.37397 | 10.07806 | 6.70408 | 7.39602E-07 | 0.00010 |
| NCAPG2 | 4.73080 | 9.79401 | 5.06320 | 7.39602E-07 | 0.00010 |
| NCAPH | 3.72854 | 8.46704 | 4.73849 | 7.39602E-07 | 0.00010 |
| ND2 | 7.33531 | 9.79583 | 2.46052 | 0.000857939 | 0.02678 |
| ND6 | 9.42631 | 11.82587 | 2.39956 | 2.95841E-06 | 0.00030 |
| NDC1 | 5.70411 | 9.17032 | 3.46621 | 0.000274392 | 0.01145 |
| NDC80 | 3.81774 | 8.18569 | 4.36795 | 2.21881E-05 | 0.00149 |
| NDST3 | 2.52650 | 7.00578 | 4.47927 | 1.4792E-06 | 0.00016 |
| NDUFA13 | 5.70236 | 7.76240 | 2.06003 | 0.00143261 | 0.04035 |
| NDUFA4 | 6.35630 | 8.54265 | 2.18635 | 0.001829776 | 0.04867 |
| NDUFAF1 | 4.57794 | 7.62639 | 3.04845 | 0.000144222 | 0.00719 |
| NDUFS6 | 6.48517 | 8.68731 | 2.20214 | 0.001829776 | 0.04867 |
| NEIL3 | 3.47032 | 9.46031 | 5.98999 | 7.39602E-07 | 0.00010 |
| NEK2 | 3.45733 | 7.62778 | 4.17045 | 1.40524E-05 | 0.00103 |
| NELL2 | 9.26069 | 3.57782 | -5.68287 | 1.4792E-06 | 0.00016 |
| NETO2 | 4.07875 | 7.24934 | 3.17058 | 0.001114581 | 0.03280 |
| NFKBIZ | 8.10534 | 3.88061 | -4.22473 | 1.40524E-05 | 0.00103 |
| NFYB | 4.92017 | 7.41322 | 2.49304 | 0.000656027 | 0.02163 |
| NIPA1 | 4.81465 | 7.50103 | 2.68638 | 0.000857939 | 0.02678 |
| NLRC5 | 8.13642 | 6.21769 | -1.91873 | 0.000656027 | 0.02163 |
| NLRP1 | 7.52729 | 4.36393 | -3.16337 | 1.40524E-05 | 0.00103 |
| NME1 | 5.65973 | 9.09637 | 3.43664 | 2.21881E-05 | 0.00149 |
| NR3C2 | 6.24078 | 3.47564 | -2.76514 | 0.000857939 | 0.02678 |
| NR4A2 | 7.59149 | 3.21613 | -4.37537 | 5.17722E-06 | 0.00045 |
| NREP | 4.22674 | 9.14211 | 4.91537 | 2.21881E-05 | 0.00149 |
| NRN1 | 3.43486 | 7.61937 | 4.18451 | 0.000201172 | 0.00897 |
| NRROS | 5.34067 | 8.63333 | 3.29265 | 0.000495534 | 0.01752 |
| NUCB2 | 7.02283 | 10.40790 | 3.38506 | 0.000495534 | 0.01752 |
| NUF2 | 3.45823 | 8.95649 | 5.49826 | 7.39602E-07 | 0.00010 |
| NUP107 | 6.21955 | 9.25728 | 3.03773 | 0.000102805 | 0.00545 |
| NUP205 | 6.83939 | 9.65894 | 2.81956 | 0.00143261 | 0.04035 |
| NUP37 | 5.53659 | 8.61602 | 3.07943 | 0.000201172 | 0.00897 |
| NUSAP1 | 3.70133 | 9.14193 | 5.44060 | 7.39602E-07 | 0.00010 |
| OGN | 2.91982 | 6.86053 | 3.94070 | 5.17722E-06 | 0.00045 |
| OIP5 | 3.18872 | 7.03834 | 3.84962 | 1.4792E-06 | 0.00016 |
| OR2A9P | 6.56398 | 4.01826 | -2.54572 | 0.00037128 | 0.01423 |
| ORC1 | 3.68800 | 7.96709 | 4.27910 | 7.39602E-07 | 0.00010 |
| ORC6 | 3.41471 | 7.97165 | 4.55694 | 1.4792E-06 | 0.00016 |
| P2RY10 | 6.88189 | 3.16512 | -3.71677 | 0.001114581 | 0.03280 |
| PAAF1 | 5.18741 | 7.72581 | 2.53840 | 0.00037128 | 0.01423 |
| PAICS | 5.81586 | 9.74477 | 3.92891 | 0.00143261 | 0.04035 |
| PARD3 | 3.18864 | 7.32363 | 4.13498 | 0.000144222 | 0.00719 |
| PARL | 6.93329 | 8.80056 | 1.86727 | 0.001829776 | 0.04867 |
| PARPBP | 2.92043 | 6.73147 | 3.81103 | 7.39602E-07 | 0.00010 |
| PBK | 2.50033 | 8.66980 | 6.16948 | 7.39602E-07 | 0.00010 |
| PBXIP1 | 7.61191 | 5.94120 | -1.67071 | 0.001829776 | 0.04867 |
| PCAT18 | 3.08561 | 5.70066 | 2.61506 | 0.000102805 | 0.00545 |
| PCCB | 6.21036 | 9.29708 | 3.08672 | 8.87523E-06 | 0.00071 |
| PCDH10 | 3.42927 | 6.08044 | 2.65118 | 0.000656027 | 0.02163 |
| PCED1B | 6.99886 | 4.16979 | -2.82907 | 0.000102805 | 0.00545 |
| PCNA | 5.54536 | 10.22924 | 4.68387 | 7.39602E-07 | 0.00010 |
| PDE4B | 5.40195 | 3.07084 | -2.33111 | 0.001829776 | 0.04867 |
| PDIA6 | 8.67259 | 10.89103 | 2.21844 | 7.39602E-07 | 0.00010 |
| PEX5L | 3.49915 | 6.55200 | 3.05286 | 0.00037128 | 0.01423 |
| PGD | 7.15050 | 10.51023 | 3.35973 | 5.17722E-06 | 0.00045 |
| PHGDH | 5.31044 | 10.04263 | 4.73219 | 7.39602E-07 | 0.00010 |
| PHKA1 | 2.98041 | 5.60966 | 2.62925 | 0.000495534 | 0.01752 |
| PHOSPHO2 | 3.68925 | 7.00005 | 3.31081 | 0.000201172 | 0.00897 |
| PIK3IP1 | 9.83274 | 4.42352 | -5.40922 | 7.39602E-07 | 0.00010 |
| PIK3R3 | 3.74707 | 7.97220 | 4.22514 | 3.32821E-05 | 0.00210 |
| PIM1 | 9.24926 | 6.21258 | -3.03668 | 1.4792E-06 | 0.00016 |
| PIM2 | 9.46197 | 5.57351 | -3.88846 | 1.4792E-06 | 0.00016 |
| PINLYP | 3.65885 | 6.62997 | 2.97112 | 0.000857939 | 0.02678 |
| PKMYT1 | 3.97740 | 7.08680 | 3.10941 | 0.000201172 | 0.00897 |
| PLCH1 | 2.81145 | 5.83731 | 3.02586 | 0.000656027 | 0.02163 |
| PLEK | 7.05277 | 3.29234 | -3.76043 | 2.95841E-06 | 0.00030 |
| PLK1 | 3.93678 | 8.96538 | 5.02860 | 7.39602E-07 | 0.00010 |
| PLK4 | 3.77446 | 8.46873 | 4.69427 | 8.87523E-06 | 0.00071 |
| PLS1 | 3.61620 | 6.75789 | 3.14170 | 0.000201172 | 0.00897 |
| PNRC1 | 10.31269 | 8.33445 | -1.97824 | 4.95534E-05 | 0.00297 |
| POC1A | 3.22558 | 6.78183 | 3.55625 | 7.17414E-05 | 0.00408 |
| POLA1 | 5.17315 | 8.92937 | 3.75621 | 5.17722E-06 | 0.00045 |
| POLA2 | 5.58834 | 8.68612 | 3.09779 | 0.001114581 | 0.03280 |
| POLE | 5.34999 | 8.50838 | 3.15839 | 0.000656027 | 0.02163 |
| POLE2 | 3.29417 | 7.17633 | 3.88216 | 4.95534E-05 | 0.00297 |
| POLQ | 2.74252 | 7.76361 | 5.02109 | 7.39602E-07 | 0.00010 |
| POLR3G | 3.76199 | 6.72447 | 2.96249 | 0.001829776 | 0.04867 |
| PPAT | 4.99533 | 8.56119 | 3.56586 | 7.39602E-07 | 0.00010 |
| PPIH | 6.20403 | 9.29938 | 3.09535 | 0.000656027 | 0.02163 |
| PPM1E | 2.98884 | 5.22832 | 2.23948 | 0.001829776 | 0.04867 |
| PPP1R1C | 2.95885 | 5.25849 | 2.29964 | 0.000656027 | 0.02163 |
| PRC1 | 4.02748 | 9.74458 | 5.71710 | 2.95841E-06 | 0.00030 |
| PRDM1 | 6.97113 | 3.58950 | -3.38163 | 0.001114581 | 0.03280 |
| PRDX3 | 6.97175 | 9.94609 | 2.97434 | 4.95534E-05 | 0.00297 |
| PRDX5 | 6.67698 | 9.21294 | 2.53595 | 0.000102805 | 0.00545 |
| PRF1 | 8.98104 | 5.25975 | -3.72129 | 7.39602E-07 | 0.00010 |
| PRIM1 | 6.38937 | 9.32642 | 2.93705 | 0.00143261 | 0.04035 |
| PRKAR2B | 3.53567 | 6.93056 | 3.39490 | 5.17722E-06 | 0.00045 |
| PRKDC | 7.64081 | 10.03422 | 2.39341 | 0.001114581 | 0.03280 |
| PRR11 | 3.43285 | 8.74806 | 5.31521 | 7.39602E-07 | 0.00010 |
| PRR5L | 6.38943 | 4.13514 | -2.25429 | 0.001829776 | 0.04867 |
| PSAT1 | 4.51294 | 9.21482 | 4.70188 | 7.39602E-07 | 0.00010 |
| PSMA4 | 7.80203 | 10.59950 | 2.79747 | 0.001114581 | 0.03280 |
| PSMB3 | 8.55220 | 10.29558 | 1.74338 | 0.00143261 | 0.04035 |
| PSMC3IP | 3.83118 | 7.79538 | 3.96420 | 4.95534E-05 | 0.00297 |
| PSMC5 | 7.62508 | 9.48236 | 1.85728 | 5.17722E-06 | 0.00045 |
| PSMD14 | 5.89196 | 9.41160 | 3.51965 | 0.000857939 | 0.02678 |
| PSRC1 | 3.93203 | 6.96018 | 3.02815 | 0.00037128 | 0.01423 |
| PTK7 | 4.44752 | 6.85917 | 2.41165 | 0.00143261 | 0.04035 |
| PTPLA | 3.36181 | 6.54434 | 3.18254 | 0.000102805 | 0.00545 |
| PTPRK | 3.70424 | 7.39173 | 3.68749 | 0.001114581 | 0.03280 |
| PTTG1 | 4.47662 | 8.45914 | 3.98252 | 1.4792E-06 | 0.00016 |
| PXDN | 3.43213 | 7.47849 | 4.04636 | 1.40524E-05 | 0.00103 |
| QPRT | 4.06923 | 7.45000 | 3.38077 | 0.000144222 | 0.00719 |
| RACGAP1 | 5.09297 | 9.32185 | 4.22887 | 7.39602E-07 | 0.00010 |
| RAD21 | 8.23048 | 10.64329 | 2.41281 | 0.000495534 | 0.01752 |
| RAD51 | 3.19991 | 7.75715 | 4.55725 | 7.39602E-07 | 0.00010 |
| RAD51AP1 | 2.97466 | 8.67352 | 5.69886 | 7.39602E-07 | 0.00010 |
| RAD54L | 3.39141 | 7.96974 | 4.57833 | 7.39602E-07 | 0.00010 |
| RANBP1 | 6.74060 | 9.58133 | 2.84074 | 7.39602E-07 | 0.00010 |
| RANP1 | 7.62573 | 10.69587 | 3.07014 | 0.00143261 | 0.04035 |
| RASGRF2 | 7.23372 | 3.26285 | -3.97087 | 0.000144222 | 0.00719 |
| RASGRP2 | 8.52558 | 6.45159 | -2.07399 | 0.000656027 | 0.02163 |
| RASSF8 | 3.26808 | 6.02220 | 2.75413 | 0.000656027 | 0.02163 |
| RBBP8 | 3.49399 | 8.68837 | 5.19438 | 7.39602E-07 | 0.00010 |
| RBFOX2 | 3.62352 | 6.30957 | 2.68605 | 0.00143261 | 0.04035 |
| RBM12B | 5.74329 | 8.14141 | 2.39812 | 0.000857939 | 0.02678 |
| RCAN3 | 8.81126 | 6.64341 | -2.16785 | 0.000144222 | 0.00719 |
| RCC1 | 5.46369 | 8.75461 | 3.29092 | 5.17722E-06 | 0.00045 |
| RCCD1 | 4.78686 | 7.41165 | 2.62480 | 0.00037128 | 0.01423 |
| RFC3 | 5.38609 | 9.72981 | 4.34373 | 7.39602E-07 | 0.00010 |
| RFX8 | 2.67310 | 5.39631 | 2.72320 | 0.000656027 | 0.02163 |
| RMI1 | 3.91672 | 7.69983 | 3.78311 | 5.17722E-06 | 0.00045 |
| RN7SKP240 | 2.45552 | 5.81371 | 3.35819 | 3.32821E-05 | 0.00210 |
| RN7SL280P | 3.01207 | 5.73516 | 2.72309 | 0.000495534 | 0.01752 |
| RN7SL288P | 5.11775 | 7.92233 | 2.80457 | 0.000201172 | 0.00897 |
| RN7SL378P | 3.13586 | 5.62765 | 2.49179 | 0.000857939 | 0.02678 |
| RN7SL381P | 3.19189 | 6.22513 | 3.03324 | 3.32821E-05 | 0.00210 |
| RN7SL390P | 2.50123 | 4.22126 | 1.72003 | 0.001114581 | 0.03280 |
| RN7SL745P | 3.13926 | 5.86399 | 2.72473 | 0.000656027 | 0.02163 |
| RNA5SP403 | 5.92712 | 7.72812 | 1.80100 | 0.001829776 | 0.04867 |
| RNASEH2A | 5.10452 | 9.07942 | 3.97489 | 5.17722E-06 | 0.00045 |
| RNF150 | 3.20765 | 5.97876 | 2.77111 | 0.00037128 | 0.01423 |
| RNF157 | 7.03783 | 4.53883 | -2.49900 | 0.001829776 | 0.04867 |
| RNF219 | 6.08059 | 8.41415 | 2.33357 | 0.000274392 | 0.01145 |
| RNU2-50P | 2.42859 | 3.99289 | 1.56430 | 0.000857939 | 0.02678 |
| RNU5D-1 | 4.68641 | 9.03959 | 4.35318 | 5.17722E-06 | 0.00045 |
| RNU6-1305P | 2.85704 | 5.12993 | 2.27289 | 0.00037128 | 0.01423 |
| RNU6-522P | 3.27118 | 7.09217 | 3.82100 | 7.39602E-07 | 0.00010 |
| RNU6-661P | 2.87742 | 5.30343 | 2.42600 | 0.000656027 | 0.02163 |
| RNVU1-15 | 6.73284 | 4.18052 | -2.55231 | 0.00037128 | 0.01423 |
| RNY4P23 | 3.44637 | 5.37297 | 1.92660 | 0.00037128 | 0.01423 |
| RPL39L | 3.24545 | 6.99858 | 3.75313 | 5.17722E-06 | 0.00045 |
| RPL7L1 | 3.43335 | 6.04260 | 2.60925 | 0.00037128 | 0.01423 |
| RRM1 | 6.27438 | 10.19653 | 3.92215 | 8.87523E-06 | 0.00071 |
| RRM2 | 3.54096 | 8.66474 | 5.12378 | 7.39602E-07 | 0.00010 |
| RUVBL2 | 5.62666 | 8.52798 | 2.90132 | 0.000274392 | 0.01145 |
| S1PR1 | 7.91788 | 4.41170 | -3.50618 | 2.21881E-05 | 0.00149 |
| SAMD13 | 2.60838 | 5.01479 | 2.40641 | 0.000201172 | 0.00897 |
| SAMD3 | 7.20620 | 2.88479 | -4.32141 | 1.40524E-05 | 0.00103 |
| SAMHD1 | 9.86059 | 4.04601 | -5.81458 | 5.17722E-06 | 0.00045 |
| SCARNA17 | 11.59299 | 8.44498 | -3.14801 | 7.39602E-07 | 0.00010 |
| SCARNA6 | 9.45238 | 6.50733 | -2.94506 | 7.39602E-07 | 0.00010 |
| SCARNA7 | 9.96369 | 8.57231 | -1.39139 | 0.001829776 | 0.04867 |
| SCCPDH | 4.85542 | 9.06926 | 4.21384 | 8.87523E-06 | 0.00071 |
| SCD | 4.16438 | 9.82998 | 5.66560 | 7.39602E-07 | 0.00010 |
| SCG5 | 2.81752 | 4.58209 | 1.76456 | 0.000656027 | 0.02163 |
| SCML2 | 3.19706 | 7.27778 | 4.08072 | 7.39602E-07 | 0.00010 |
| SCML4 | 6.79330 | 4.26305 | -2.53025 | 0.000274392 | 0.01145 |
| SCN3A | 2.56335 | 6.03400 | 3.47064 | 0.001829776 | 0.04867 |
| SESN3 | 8.61423 | 4.55819 | -4.05604 | 1.4792E-06 | 0.00016 |
| SESTD1 | 4.15087 | 7.37048 | 3.21961 | 0.000857939 | 0.02678 |
| SGOL1 | 3.00588 | 7.61383 | 4.60795 | 7.39602E-07 | 0.00010 |
| SGOL2 | 3.40488 | 7.13043 | 3.72555 | 0.000201172 | 0.00897 |
| SHCBP1 | 3.58845 | 9.48285 | 5.89440 | 7.39602E-07 | 0.00010 |
| SKA1 | 2.74986 | 6.57843 | 3.82857 | 5.17722E-06 | 0.00045 |
| SKA3 | 3.01014 | 8.17235 | 5.16221 | 7.39602E-07 | 0.00010 |
| SLC16A1 | 4.77785 | 8.75179 | 3.97394 | 7.17414E-05 | 0.00408 |
| SLC1A4 | 3.96682 | 7.78273 | 3.81591 | 0.000201172 | 0.00897 |
| SLC29A1 | 4.15127 | 8.19127 | 4.04000 | 5.17722E-06 | 0.00045 |
| SLC2A3 | 9.79573 | 7.75398 | -2.04175 | 0.000495534 | 0.01752 |
| SLC7A1 | 5.97271 | 9.14991 | 3.17719 | 0.00143261 | 0.04035 |
| SLC7A11 | 2.57473 | 7.12320 | 4.54848 | 7.39602E-07 | 0.00010 |
| SLC7A5 | 5.03514 | 9.78833 | 4.75319 | 5.17722E-06 | 0.00045 |
| SLFN12L | 8.92905 | 5.68798 | -3.24107 | 0.000102805 | 0.00545 |
| SLIT1 | 3.67153 | 7.29585 | 3.62432 | 0.000274392 | 0.01145 |
| SMC2 | 4.71068 | 9.17327 | 4.46259 | 7.39602E-07 | 0.00010 |
| SMC4 | 6.12383 | 9.45326 | 3.32943 | 0.000857939 | 0.02678 |
| SMCO4 | 3.51052 | 6.42221 | 2.91169 | 0.000857939 | 0.02678 |
| SNORD94 | 9.63737 | 7.52360 | -2.11377 | 2.21881E-05 | 0.00149 |
| SNRPD1 | 5.39417 | 8.63685 | 3.24268 | 0.000144222 | 0.00719 |
| SNRPD3 | 7.35563 | 9.69371 | 2.33808 | 0.000274392 | 0.01145 |
| SORD | 4.69859 | 8.31142 | 3.61283 | 8.87523E-06 | 0.00071 |
| SORL1 | 9.49330 | 5.90079 | -3.59251 | 4.95534E-05 | 0.00297 |
| SORT1 | 3.32340 | 7.34884 | 4.02544 | 0.000274392 | 0.01145 |
| SOX4 | 4.41806 | 8.64062 | 4.22255 | 1.40524E-05 | 0.00103 |
| SPAG5 | 3.77549 | 8.65392 | 4.87843 | 1.4792E-06 | 0.00016 |
| SPC24 | 3.16660 | 7.66967 | 4.50307 | 7.39602E-07 | 0.00010 |
| SPC25 | 2.29997 | 7.46091 | 5.16094 | 7.39602E-07 | 0.00010 |
| SPNS3 | 3.91619 | 8.21822 | 4.30202 | 0.000201172 | 0.00897 |
| SPOCK2 | 9.51837 | 4.82709 | -4.69128 | 7.39602E-07 | 0.00010 |
| SPRED2 | 3.58915 | 6.69734 | 3.10819 | 0.000656027 | 0.02163 |
| SRGN | 10.04152 | 7.82871 | -2.21281 | 0.001114581 | 0.03280 |
| SSRP1 | 5.42571 | 7.86660 | 2.44089 | 0.000102805 | 0.00545 |
| STARD4 | 5.28653 | 8.34464 | 3.05811 | 0.000495534 | 0.01752 |
| STAT4 | 8.65609 | 4.44570 | -4.21040 | 0.000656027 | 0.02163 |
| STIL | 3.34518 | 7.63500 | 4.28982 | 0.000201172 | 0.00897 |
| STIP1 | 6.06503 | 9.34538 | 3.28035 | 0.001114581 | 0.03280 |
| STK3 | 3.59452 | 6.53318 | 2.93866 | 0.001829776 | 0.04867 |
| STK33 | 2.73812 | 5.23198 | 2.49386 | 0.001114581 | 0.03280 |
| STMN1 | 4.44543 | 7.53381 | 3.08838 | 0.000495534 | 0.01752 |
| STRBP | 5.11366 | 8.19208 | 3.07842 | 0.000495534 | 0.01752 |
| STRIP2 | 3.08185 | 6.07849 | 2.99664 | 3.32821E-05 | 0.00210 |
| STXBP5L | 2.51173 | 5.16722 | 2.65549 | 0.000201172 | 0.00897 |
| SUCLA2 | 6.34698 | 9.05828 | 2.71130 | 0.00037128 | 0.01423 |
| SULT1B1 | 6.07502 | 2.97166 | -3.10336 | 0.001114581 | 0.03280 |
| SUN2 | 9.43367 | 7.77379 | -1.65987 | 0.000857939 | 0.02678 |
| SUPT16H | 7.56083 | 10.34967 | 2.78884 | 0.001829776 | 0.04867 |
| SUV39H2 | 4.97875 | 8.14487 | 3.16611 | 0.000274392 | 0.01145 |
| SV2A | 4.16399 | 7.10910 | 2.94511 | 0.000495534 | 0.01752 |
| SYTL3 | 6.44418 | 3.79025 | -2.65394 | 0.001114581 | 0.03280 |
| TACC3 | 6.16703 | 8.44603 | 2.27900 | 0.00143261 | 0.04035 |
| TAGAP | 8.99397 | 4.87401 | -4.11996 | 1.4792E-06 | 0.00016 |
| TAL1 | 3.59566 | 6.58504 | 2.98938 | 0.000857939 | 0.02678 |
| TANC1 | 3.29869 | 6.92734 | 3.62866 | 1.40524E-05 | 0.00103 |
| TARS | 6.94421 | 10.33679 | 3.39257 | 7.39602E-07 | 0.00010 |
| TC2N | 9.68547 | 3.46562 | -6.21985 | 7.39602E-07 | 0.00010 |
| TCF19 | 4.46919 | 8.08141 | 3.61222 | 1.4792E-06 | 0.00016 |
| TCP1 | 9.48495 | 11.87929 | 2.39434 | 2.95841E-06 | 0.00030 |
| TEX30 | 3.74687 | 7.28280 | 3.53594 | 0.000144222 | 0.00719 |
| TFDP2 | 6.66147 | 9.96848 | 3.30701 | 0.000656027 | 0.02163 |
| TFRC | 6.76416 | 10.37232 | 3.60815 | 0.000102805 | 0.00545 |
| TGFBR2 | 9.46469 | 7.03953 | -2.42516 | 0.00037128 | 0.01423 |
| TGFBR3 | 8.01273 | 3.54365 | -4.46908 | 7.39602E-07 | 0.00010 |
| THEM4 | 7.13461 | 3.48106 | -3.65355 | 2.21881E-05 | 0.00149 |
| TICRR | 3.28330 | 8.15540 | 4.87211 | 7.39602E-07 | 0.00010 |
| TIGIT | 6.60953 | 3.13748 | -3.47205 | 2.95841E-06 | 0.00030 |
| TIMELESS | 4.12282 | 8.60182 | 4.47901 | 7.39602E-07 | 0.00010 |
| TIMM17A | 4.77063 | 7.70584 | 2.93521 | 0.000857939 | 0.02678 |
| TIPIN | 4.67384 | 7.92561 | 3.25177 | 0.000656027 | 0.02163 |
| TM6SF1 | 3.01730 | 7.72861 | 4.71131 | 7.39602E-07 | 0.00010 |
| TMBIM1 | 8.40869 | 5.40189 | -3.00680 | 0.000102805 | 0.00545 |
| TMEM194A | 6.96034 | 9.36515 | 2.40482 | 0.000857939 | 0.02678 |
| TMEM2 | 7.92237 | 5.74187 | -2.18049 | 0.000144222 | 0.00719 |
| TMEM204 | 8.34290 | 5.23111 | -3.11178 | 0.000102805 | 0.00545 |
| TMEM237 | 3.78487 | 7.21725 | 3.43238 | 0.000201172 | 0.00897 |
| TMEM63A | 9.36971 | 6.72711 | -2.64260 | 0.000144222 | 0.00719 |
| TMEM67 | 4.09270 | 6.76591 | 2.67320 | 0.00037128 | 0.01423 |
| TNFRSF10A | 7.46143 | 3.88418 | -3.57725 | 7.39602E-07 | 0.00010 |
| TNFRSF10D | 7.09434 | 4.29114 | -2.80320 | 1.40524E-05 | 0.00103 |
| TNFRSF21 | 3.44141 | 6.28047 | 2.83907 | 0.000274392 | 0.01145 |
| TNFRSF25 | 8.60322 | 6.15999 | -2.44323 | 1.40524E-05 | 0.00103 |
| TNFSF8 | 8.67855 | 4.82568 | -3.85287 | 4.95534E-05 | 0.00297 |
| TNIK | 8.03505 | 5.30119 | -2.73386 | 0.001829776 | 0.04867 |
| TOP2A | 3.78808 | 10.26607 | 6.47799 | 7.39602E-07 | 0.00010 |
| TPX2 | 3.33070 | 9.44874 | 6.11803 | 7.39602E-07 | 0.00010 |
| TRABD2A | 8.43598 | 5.10826 | -3.32772 | 1.4792E-06 | 0.00016 |
| TRADD | 8.05471 | 6.64401 | -1.41070 | 0.001829776 | 0.04867 |
| TRAF1 | 6.65309 | 3.87788 | -2.77521 | 0.000102805 | 0.00545 |
| TRAJ41 | 6.86119 | 3.69592 | -3.16527 | 0.001114581 | 0.03280 |
| TRIM22 | 8.93612 | 5.18260 | -3.75352 | 3.32821E-05 | 0.00210 |
| TRIP13 | 3.21399 | 7.98282 | 4.76884 | 7.39602E-07 | 0.00010 |
| TROAP | 3.70303 | 7.71397 | 4.01094 | 2.21881E-05 | 0.00149 |
| TRPS1 | 5.68433 | 2.85868 | -2.82565 | 0.000656027 | 0.02163 |
| TSC22D3 | 8.59085 | 4.71466 | -3.87619 | 1.40524E-05 | 0.00103 |
| TSPAN13 | 3.06736 | 5.55904 | 2.49168 | 0.00037128 | 0.01423 |
| TSPAN18 | 6.67547 | 4.18752 | -2.48795 | 0.000274392 | 0.01145 |
| TSPAN3 | 6.78429 | 9.26160 | 2.47731 | 3.32821E-05 | 0.00210 |
| TSPAN7 | 3.37675 | 10.12094 | 6.74419 | 0.000144222 | 0.00719 |
| TTC26 | 3.37684 | 6.12275 | 2.74591 | 0.00143261 | 0.04035 |
| TTI2 | 5.01824 | 7.85914 | 2.84090 | 0.000495534 | 0.01752 |
| TTK | 2.64817 | 8.46005 | 5.81188 | 7.39602E-07 | 0.00010 |
| TUSC3 | 3.27493 | 6.34445 | 3.06952 | 0.000201172 | 0.00897 |
| TXK | 8.64240 | 4.17665 | -4.46575 | 2.95841E-06 | 0.00030 |
| TXN | 5.26608 | 9.66016 | 4.39408 | 5.17722E-06 | 0.00045 |
| TXNIP | 12.57404 | 7.88801 | -4.68603 | 1.4792E-06 | 0.00016 |
| TXNRD1 | 6.37279 | 9.53204 | 3.15924 | 7.39602E-07 | 0.00010 |
| TYMS | 4.09714 | 10.79296 | 6.69582 | 7.39602E-07 | 0.00010 |
| UBE2C | 4.01920 | 7.93071 | 3.91151 | 5.17722E-06 | 0.00045 |
| UBE2MP1 | 4.46849 | 7.83333 | 3.36484 | 2.95841E-06 | 0.00030 |
| UBE2T | 3.94234 | 8.78634 | 4.84400 | 7.39602E-07 | 0.00010 |
| UCK2 | 5.08237 | 9.07572 | 3.99335 | 1.40524E-05 | 0.00103 |
| ULBP1 | 3.40049 | 6.20035 | 2.79986 | 0.00037128 | 0.01423 |
| UNG | 4.64826 | 7.45028 | 2.80202 | 0.00143261 | 0.04035 |
| UQCRC1 | 5.40950 | 8.13298 | 2.72349 | 0.000495534 | 0.01752 |
| UQCRHL | 6.54402 | 9.31192 | 2.76789 | 7.39602E-07 | 0.00010 |
| USP1 | 6.24792 | 8.94187 | 2.69395 | 0.001829776 | 0.04867 |
| USP12 | 4.90914 | 7.61567 | 2.70654 | 0.000201172 | 0.00897 |
| USP44 | 4.07912 | 8.18812 | 4.10900 | 1.4792E-06 | 0.00016 |
| VANGL1 | 3.80550 | 7.34811 | 3.54261 | 0.000656027 | 0.02163 |
| VASH2 | 3.65627 | 6.82356 | 3.16729 | 0.000274392 | 0.01145 |
| VAT1 | 5.72469 | 9.01427 | 3.28958 | 0.000495534 | 0.01752 |
| VDAC1P4 | 4.05001 | 7.07266 | 3.02266 | 0.00143261 | 0.04035 |
| VLDLR | 3.18457 | 7.88133 | 4.69676 | 2.21881E-05 | 0.00149 |
| VRK1 | 6.39794 | 9.50690 | 3.10896 | 0.001114581 | 0.03280 |
| VSIG1 | 7.45670 | 2.76381 | -4.69290 | 3.32821E-05 | 0.00210 |
| WDHD1 | 4.03969 | 8.99320 | 4.95351 | 7.39602E-07 | 0.00010 |
| WDR12 | 5.55167 | 8.68309 | 3.13142 | 3.32821E-05 | 0.00210 |
| WDR34 | 4.77188 | 7.68792 | 2.91604 | 0.00037128 | 0.01423 |
| WDR62 | 3.86275 | 7.05607 | 3.19333 | 0.00037128 | 0.01423 |
| WDR77 | 6.71581 | 8.74708 | 2.03127 | 0.000495534 | 0.01752 |
| WEE1 | 4.15111 | 8.02337 | 3.87226 | 0.00037128 | 0.01423 |
| WHSC1 | 4.83695 | 8.58153 | 3.74458 | 0.000201172 | 0.00897 |
| WT1 | 3.86737 | 6.96215 | 3.09478 | 0.000102805 | 0.00545 |
| XRCC2 | 3.39679 | 8.66023 | 5.26344 | 7.39602E-07 | 0.00010 |
| YARS | 6.69240 | 9.36108 | 2.66867 | 0.00143261 | 0.04035 |
| YPEL2 | 7.08859 | 4.28810 | -2.80049 | 0.000144222 | 0.00719 |
| YPEL3 | 7.17739 | 4.53475 | -2.64264 | 0.000102805 | 0.00545 |
| YWHAE | 7.02738 | 10.73196 | 3.70457 | 0.000857939 | 0.02678 |
| ZAK | 3.10060 | 6.29206 | 3.19146 | 0.000495534 | 0.01752 |
| ZBED8 | 2.33886 | 4.47137 | 2.13251 | 0.000201172 | 0.00897 |
| ZBTB38 | 8.18038 | 3.65229 | -4.52810 | 1.4792E-06 | 0.00016 |
| ZC3HAV1L | 3.95476 | 7.66222 | 3.70747 | 1.4792E-06 | 0.00016 |
| ZGRF1 | 4.11200 | 7.81358 | 3.70158 | 0.000495534 | 0.01752 |
| ZNF322 | 4.19602 | 6.70709 | 2.51107 | 0.001829776 | 0.04867 |
| ZNF443 | 2.96692 | 5.81710 | 2.85018 | 3.32821E-05 | 0.00210 |
| ZNF670 | 3.25858 | 6.36953 | 3.11095 | 0.000102805 | 0.00545 |
| ZNF695 | 2.89105 | 6.02734 | 3.13629 | 0.000495534 | 0.01752 |
| ZNF711 | 3.66212 | 7.77609 | 4.11397 | 0.000201172 | 0.00897 |
| ZNF730 | 2.77438 | 6.64076 | 3.86638 | 2.21881E-05 | 0.00149 |
| ZNF788 | 2.60409 | 5.98470 | 3.38061 | 0.000102805 | 0.00545 |
| ZNF833P | 2.86428 | 4.79310 | 1.92881 | 0.001114581 | 0.03280 |
| ZNRF1 | 4.12013 | 7.47890 | 3.35878 | 4.95534E-05 | 0.00297 |
| ZWINT | 4.15047 | 8.38800 | 4.23754 | 8.87523E-06 | 0.00071 |
